# Ciclopirox and bortezomib synergistically inhibits glioblastoma multiforme growth via simultaneously enhancing JNK/p38 MAPK and NF-κB signaling

**DOI:** 10.1038/s41419-021-03535-9

**Published:** 2021-03-05

**Authors:** Zhipeng Su, Shengnan Han, Qiumei Jin, Ningning Zhou, Junwan Lu, Fugen Shangguan, Shiyi Yu, Yongzhang Liu, Lu Wang, Jianglong Lu, Qun Li, Lin Cai, Chengde Wang, Xiaohe Tian, Lingyan Chen, Weiming Zheng, Bin Lu

**Affiliations:** 1 Department of Neurosurgery, The First Affiliated Hospital of Wenzhou Medical University, Wenzhou, 325000 Zhejiang China; 2 Protein Quality Control and Diseases Laboratory, Key Laboratory of Medical Genetics of Zhejiang Province, Key Laboratory of Laboratory Medicine, Ministry of Education of China School of Laboratory Medicine and Life Sciences, Wenzhou Medical University, Wenzhou, 325035 Zhejiang China; 3 Department of Pathology, The Second Hospital of Jiaxing, Jiaxing University, Jiaxing, 314000 China; 4 Huaxi MR Research Center (HMRRC), Department of Radiology, Functional and molecular imaging Key Laboratory of Sichuan Province, West China Hospital of Sichuan University, Chengdu, 610041 China

**Keywords:** Cancer metabolism, Drug development

## Abstract

Ciclopirox (CPX) is an antifungal drug that has recently been reported to act as a potential anticancer drug. However, the effects and underlying molecular mechanisms of CPX on glioblastoma multiforme (GBM) remain unknown. Bortezomib (BTZ) is the first proteasome inhibitor-based anticancer drug approved to treat multiple myeloma and mantle cell lymphoma, as BTZ exhibits toxic effects on diverse tumor cells. Herein, we show that CPX displays strong anti-tumorigenic activity on GBM. Mechanistically, CPX inhibits GBM cellular migration and invasion by reducing N-Cadherin, MMP9 and Snail expression. Further analysis revealed that CPX suppresses the expression of several key subunits of mitochondrial enzyme complex, thus leading to the disruption of mitochondrial oxidative phosphorylation (OXPHOS) in GBM cells. In combination with BTZ, CPX promotes apoptosis in GBM cells through the induction of reactive oxygen species (ROS)-mediated c-Jun N-terminal kinase (JNK)/p38 mitogen-activated protein kinase (MAPK) signaling. Moreover, CPX and BTZ synergistically activates nuclear factor kappa B (NF-κB) signaling and induces cellular senescence. Our findings suggest that a combination of CPX and BTZ may serve as a novel therapeutic strategy to enhance the anticancer activity of CPX against GBM.

## Introduction

Ciclopirox (CPX), 6-cyclohexyl-1-hydroxy-4-methylpyridin-2-one, is a synthetic hydroxypyridone derivative^1^. As Batrafen, Loprox, or Penlac, CPX olamine is currently used topically to treat fungal infections of the skin^1^. CPX is an iron chelator that forms complexes with trivalent metal cations such as Fe^3+^, leading to the inhibition of metal-dependent enzymatic activity, including that of peroxidase. Intracellularly, peroxidase plays a protective role in the decomposition of H_2_O_2_ and the reduction of organic peroxides^2^. CPX can induce cell death in a variety of cancers^3–6^; for example, CPX inhibits tumor growth in xenografts of the human breast cancer cell line, MDA-MB231^3^. Our previous study reported that CPX can activate PERK-dependent endoplasmic reticulum (ER) stress to induce cell death and overcome chemoresistance in colorectal cancer cells^7^. In a phase I clinical trial, CPX showed efficacy and tolerability in the treatment of patients with relapsed or refractory hematologic malignancies^8^. However, the roles and underlying mechanisms of CPX in glioblastoma multiforme (GBM) remain unknown.

Bortezomib (BTZ, Velcade) is the first proteasome inhibitor-based anticancer drug approved by the US FDA to treat multiple myeloma and mantle cell lymphoma^9^. Phase I clinical trials showed that BTZ can be used as a chemosensitizer in refractory and relapsed acute lymphoblastic leukemia^10,11^. Subsequent phase I and phase II clinical trials for the treatment of childhood leukemia and lymphoma demonstrated that BTZ can restore the chemosensitivity of leukemia cells to conventional drugs^12^. In addition, more than 40 early clinical trials, comprising approximately two dozen targeted small molecules and BTZ, are currently being evaluated^13^. Mechanistically, BTZ may inhibit the activation of nuclear factor kappa B (NF-κB) signaling by regulating the stabilization of I-kappa-B-alpha (IκBα) in T-cell acute lymphoblastic leukemia^14,15^. In contrast, the activation of NF-κB is associated with temozolomide (TMZ)-induced senescence in GBM cells^16^.

GBM is highly invasive and often infiltrates surrounding normal brain tissue, making it almost impossible to completely remove the tumor. In the present study, we investigated whether BTZ can increase the sensitivity of GBM to CPX and evaluated the synergistic effects of CPX and BTZ in GBM cells. We demonstrated in GBM cells that CPX blunted expressions of N-Cadherin, MMP9 and Snail, which subsequently led to inhibition of the cell migration and invasion. We further revealed that CPX impaired mitochondrial oxidative phosphorylation (OXPHOS) through inhibiting key subunits of the mitochondrial electron transport chain enzyme complexes, and thereby suppressed cell growth and induced apoptosis. Importantly, we uncover a novel molecular mechanism by which CPX combined with BTZ exhibited synergistic effects in promoting GBM cellular death and senescence by simultaneously enhancing the activation of reactive oxygen species (ROS)-mediated JNK/p38 MAPK and NF-κB pathways. Our findings provide a potential novel strategy for the use of CPX and BTZ to improve the survival of GBM patients, as well as to reduce the cost of treatments.

## Materials and methods

### Cell culture

Human glioblastoma cell lines U251, SF126, A172, and U118 were obtained from the Cell Bank of Shanghai Institute of Cell Biology (Shanghai, China) and maintained in high-glucose DMEM (Dulbecco’s Modified Eagle Medium) supplemented with 10% fetal bovine serum (FBS; Thermo Fisher Scientific, NY, USA) and 1% penicillin/streptomycin. Cells were incubated at 37 °C in a humidified incubator with 5% CO_2_. All of the cell lines were authenticated and confirmed to be mycoplasma-free by the Cell Bank of the Chinese Academy of Sciences before use. We also routinely tested all the cell lines and confirmed them to be mycoplasma-free by a PCR-based assay during the present study.

### Reagents

Ciclopirox olamine (CPX) was purchased from Dibo Chemical Technology Limited Company (Shanghai, China). BTZ was obtained from Targetmol (MA, USA). Antimycin A, carbonylcyanide-p-trifluoromethoxyphenylhydrazone, oligomycin, and rotenone were purchased from Sigma Aldrich (MO, USA). The Cell Mitochondria Isolation Kit, crystal violet, MTT cell proliferation and cytotoxicity kits, N-acetyl cysteine (NAC), and Nuclear and Cytoplasmic Protein Extraction Kits were purchased from Beyotime Biotechnology (Shanghai, China). Annexin V-fluorescein isothiocyanate (FITC)/propidium iodide (PI) apoptosis detection kits were purchased from BD Biosciences (CA, USA), Pierce BCA Protein Assay Kits and TRIzol reagent from Thermo Fisher Scientific (MA, USA), and Protease (cOmplete Mini) and phosphatase-inhibitor cocktail tablets (PhosSTOP) from Roche Applied Science (Penzberg, Germany).

### Cell viability and proliferation assays

Cells were seeded in 96-well plates at a density of 5 × 10^3^ cells per well and the indicated concentrations (DMSO, 5, 10, 20, 40, 80, 160, and 320 μM) of CPX were added on the next day for another 48 h. Cell viability was measured by MTT cell proliferation and cytotoxicity kits according to the manufacturer’s instructions. For proliferation assays, cells were seeded in 96-well plates at a density of 3 × 10^3^ cells per well and treated on the next day with a various concentrations (20, 40, and 80 μM for U251 cells; 10, 20, and 40 μM for SF126 cells) of CPX or vehicle (DMSO) alone for 0, 1, 2, 3, and 4 days. The proliferation of GBM cell proliferation was measured using MTT assays.

### Colony formation assay

Colony formation assay is widely used to test the ability of a single cell to grow into a colony, which enables us to predict tumor cell sensitivity to cytostatic drugs. We performed the colony formation assay as described previously^17^. Briefly, GBM cells were seeded at a density of 1 × 10^3^ cells per well into six-well plates and were cultured at 37 °C in a humidified incubator with 5% CO_2_. After colonies became visible, the medium was replaced with fresh DMEM containing vehicle (DMSO), CPX, BTZ, or a combination of CPX and BTZ. Cells were incubated for another 5–7 days and then fixed with methanol for 20 min at room temperature. Colonies were stained with 0.1% crystal violet for 20 min at room temperature and imaged and quantified using ImageJ software. The BTZ concentration used (24 nM) was bsed on the published work by Comba A and Vlachostergios PJ^18,19^.

### Transwell cell migration and invasion assays

Cell migration and invasion were analyzed using a Transwell assay (24-well plates; 8-μm pore-size insert; Corning Inc., ME, USA) as described^17^. Cultures were treated with CPX for 48 h. Migrated cells were stained with 0.1% crystal violet then imaged and quantified.

### RNA preparation and quantitative real-time polymerase chain reaction (qRT-PCR)

Total RNA was extracted from cultured cells or tissue samples using Trizol reagent (ThermoFischer, NY, USA) following the manufacturer’s protocol. qRT-PCR was carried out as described previously^20^. Primer sequences used for qRT-PCR are listed in Supplementary Table S1.

### Isolation of cytoplasmic, nuclear, and mitochondrial fractions

The Nuclear and Cytoplasmic Protein Extraction Kit and Cell Mitochondria Isolation Kit (Beyotime Biotechnology, Shanghai, China) were used to separate cytosolic, mitochondrial, and nuclear fractions according to the manufacturer’s protocols.

### Protein extraction and immunoblot analysis

Total proteins from lysed cells or tumor tissues were extracted using RIPA buffer (50 mM Tris-HCl, pH 7.4, 1.0% Triton X-100, 1% sodium deoxycholate, 0.1% SDS, 150 mM NaCl) containing protease and phosphatase inhibitors (1 mM NaF and 1 mM Na_3_VO_4_) on ice. Samples were centrifuged at 12,000 *g* for 20 min at 4 °C, after which the supernatants were collected. Protein concentrations were determined using the Pierce BCA protein assay kit, and immunoblot analysis was performed using a standard protocol as described previously^20^. Optical densities of protein bands were determined using ImageJ. The details of the primary antibodies are provided in Supplementary Table S2.

### Oxidative phosphorylation assay

Measurements of intact cellular oxygen consumption rates (OCRs) of A172, U118, U251, and SF126 cells were performed with a Seahorse XF-96 Extracellular Flux Analyzer (Agilent Technologies Inc., North Billerica, MA), as described^20^.

### Flow cytometry analysis for detection of ROS and apoptosis

For the ROS assay, U251 and SF126 cells were incubated with the indicated concentrations of CPX, NAC, or BTZ for 48 h. Cells were harvested, washed with pre-cooled PBS, and stained with DCFH-DA (for intracellular ROS) or MitoSOX (for mitochondrial ROS) at a final concentration of 10 µM in DMEM at 37 °C for 30 min. The cells were washed twice with pre-cooled DMEM and subjected to flow cytometry analysis using a BD Accuri C6 plus flow cytometer (BD Biosciences, NJ, USA). For the apoptotic assay, 6 × 10^6^ cells were plated in 6-cm culture dishes and cultured overnight at 37 °C with 5% CO_2_ in DMEM. After attachment, the cells were treated with CPX (20 μM), BTZ (24 nM) or a combination of both drugs for 24 h. Cells were harvested, stained with annexin V-FITC/PI (BD, San Jose, CA) in the dark at room temperature for 20 min, and then subjected to FACS analysis.

### Immunofluorescence

Immunofluorescent analysis was carried out as previously described^17^.

### In vivo studies

U251 or U118 cells (1 × 10^7^ cells each) in 100 μl of PBS were subcutaneously injected into the dorsal-left flank of 6-week-old male BALB/c athymic nude mice (SLAC, Shanghai, China). After tumors reached ~300–400 mm^3^ in volume, mice were randomized into two groups (six mice per group) for treatment with vehicle control (0.9% NaCl) or CPX. The mice were then administered an intraperitoneal injection of CPX (dissolved in 0.9% NaCl, 20 mg/kg for U251 cells and 15 mg/kg for U118 cells) or (0.9% NaCl), once a day for 12 days. To study the effects of the combination of CPX and BTZ on GBM cell (U251 and SF126) growth in vivo, the tumor-bearing mice were randomized into four groups (five mice per group) following administered an intraperitoneal injection of vehicle control (0.9% NaCl), CPX (5 mg/kg), a combination of CPX (5 mg/kg) and BTZ (0.4 mg/kg), and BTZ (0.4 mg/kg), once a day for 14 days. Tumor growth was monitored every two days by measuring the length (L) and width (W) of the tumor, using a digital caliper, and tumor volume was calculated according to the following formula: volume = 1/2 × *L* × *W*^2^. Mouse body weights were also measured every 2 days. All of the mice were sacrificed after 14 days, after which tumors were dissected, imaged, and weighed. The animal experiments were approved by the Institutional Animal Care and Use Committee at the University Laboratory Animal Research of Wenzhou Medical University, and were carried out in accordance with institutional guidelines.

### Statistical analysis

Statistical analyses were performed using SPSS Standard version 16.0 (SPSS Inc., IL, USA) and graphs were plotted via GraphPad Prism 5 (GraphPad Software, CA, USA). Statistical differences between two groups were analyzed using Student’s t tests. Data are reported as means ± standard deviations (SDs) of three independent experiments. Statistical significance was considered at  *p* < 0.05, and denoted as follows in the figures: *p < 0.05; **p < 0.01; ***p < 0.001.

## Results

### CPX inhibits GBM cell growth in vitro and in vivo

**Fig. 1:**
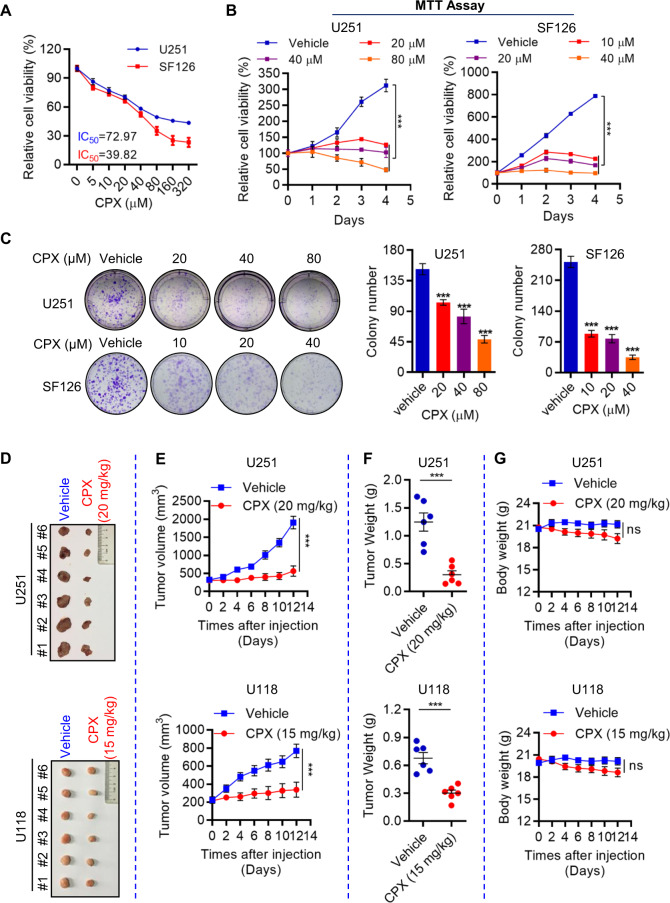
CPX suppresses GBM cell growth in vitro and in vivo. **A** The IC_50_ of U251 and SF126 cells treated with CPX for 48 h was evaluated using MTT cellular proliferation and cytotoxicity assay kits. **B** Proliferation of U251 and SF126 cells treated with CPX for four days was measured by MTT cellular proliferation and cytotoxicity assay kits. Data represent mean ± SD (*n* = 3, ***p < 0.001). **C** Colony formation assays of U251 and SF126 cells treated with or without CPX for 48 h. Representative images of colony formation assays are shown (left panel). Colony number counts (right panel) are presented as the mean ± SD (*n* = 3, ***p < 0.001). **D**–**G** Nude mice bearing U251 and U118 cell tumors (*n* = 6, each cell line) were injected with either CPX or 0.9% NaCl. After 12 days of once-daily injections, the mice were sacrificed. Representative dissected tumors are shown (**D**). Tumor volumes were measured at the indicated intervals (**E**) and dissected tumors were weight at the end of the 14-day period (**F**). The body weight of mice treated with CPX or 0.9% NaCl was measured at the indicated intervals. Data represent mean ± SD (*n* = 6; ***p < 0.001. ns, not significant) (**G**).

To investigate the effect of CPX on GBM cells in vitro, we first evaluated the cytotoxicity of CPX in a panel of GBM cells (U251, SF126, A172, and U118 cells; Fig. 1A and Supplementary Fig. S1a). The IC_50_ values measured ranged from 34–74 µM. In cell proliferation assays, CPX rapidly suppressed GBM cell growth in culture (Fig. 1B and Supplementary Fig. S1B). In clonogenic assays, CPX significantly inhibited colony formation in GBM cells in a dose-dependent manner (Fig. 1C and Supplementary Fig. S1c).

We used a mouse xenograft model to examine the effect of CPX on GBM cell growth in vivo. CPX dramatically suppressed the growth of U251 and U118 cell tumors as compared to vehicle control in vivo (Fig. 1D, E). Tumor weight decreased significantly in the CPX-treated group (Fig. 1F) without notable loss of body weight (Fig. 1G).

Taken together, these results demonstrate that CPX inhibits GBM cell growth in vivo and in vitro at concentrations exhibiting only minor toxicity.

### CPX blocks GBM cellular migration and invasion in vitro

**Fig. 2:**
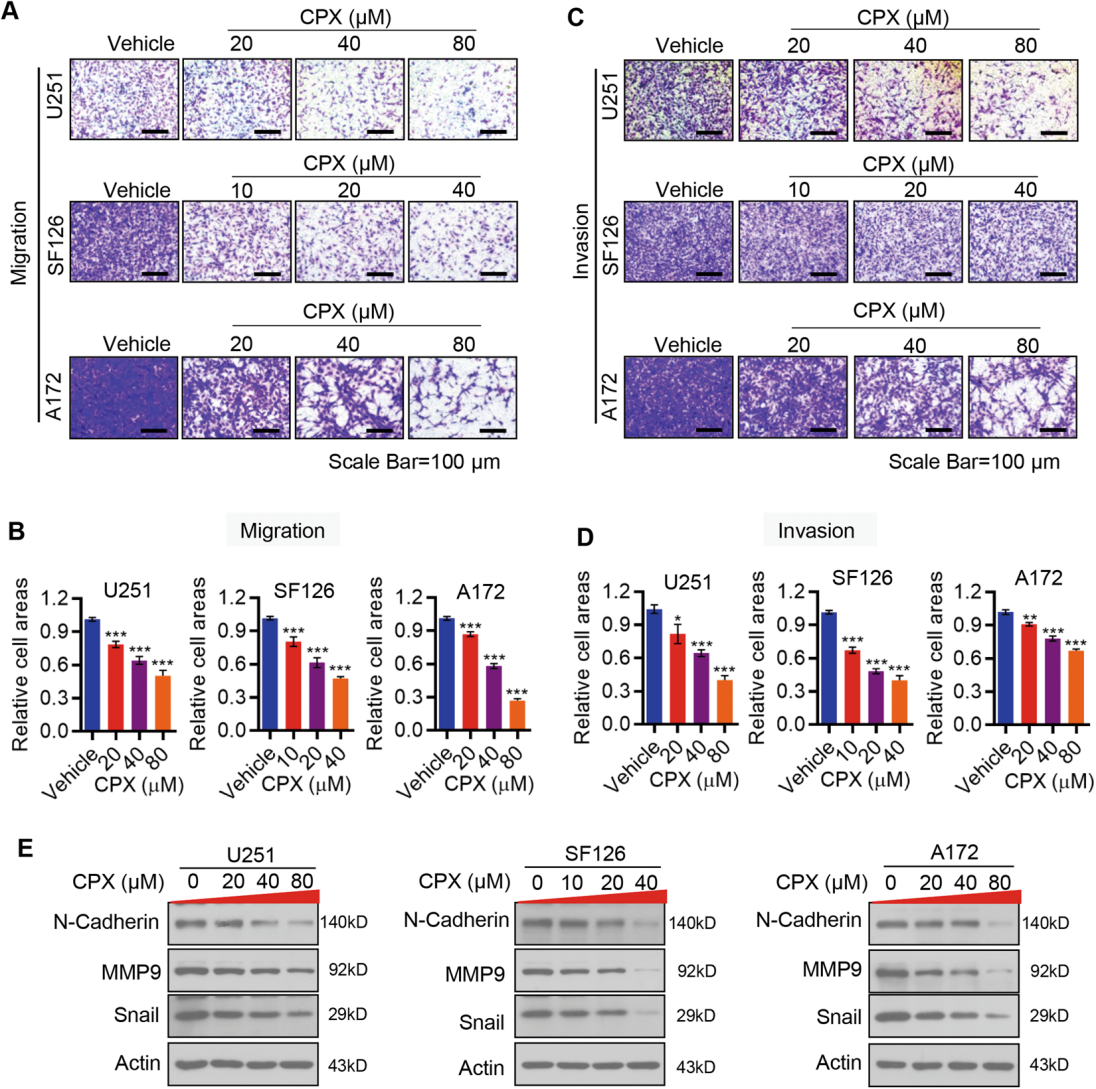
CPX inhibits GBM cell migration and invasion. **A**, **B** Representative image of transwell migration (**A**) and quantitation of cell migration (**B**) of U251, SF126, and A172 cells. Migrated cells were stained with 0.1% crystal violet and were then imaged and quantified. Data represent mean ± SD (*n* = 3; ***p < 0.001). **C**, **D** Representative images of invasion (**C**) and quantitation of cellular invasion (**D**) of U251, SF126, and A172 cells. Invaded cells were stained with 0.1% crystal violet and were then imaged and quantified. Data represent mean ± SD (*n* = 3; *p < 0.05; **p < 0.01; ***p < 0.001). **E** U251, SF126, and A172 cells were treated with a range of CPX concentrations for 48 h, followed by immunoblot analysis with the indicated antibodies.

Tumor metastasis and invasion contribute to the high mortality rate and poor prognosis of GBM. To explore the impact of CPX on GBM metastasis, we used transwell assay to evaluate the effect of CPX on cell migration and invasion. Our data show that CPX treatment markedly inhibited migration of U251, SF126 and A172 cells in a dose-dependent manner (Fig. 2A, B). Similarly, CPX treatment dramatically inhibited the invasive potential of GBM cells in a dose-dependent manner (Fig. 2C, D). Thus, CPX inhibited cell migration and invasion, two critical facets of many key biological processes of cancer cells that are essential for metastasis, which is the major cause of death in cancer patients.

At the molecular level, immunoblotting revealed that CPX treatment of GBM cells significantly reduced the expression of two pro-metastatic proteins, N-Cadherin and Snail, and of the pro-invasive protein, MMP9 (Fig. 2E).

### CPX impairs mitochondrial respiration and induces ROS production in GBM cells

**Fig. 3:**
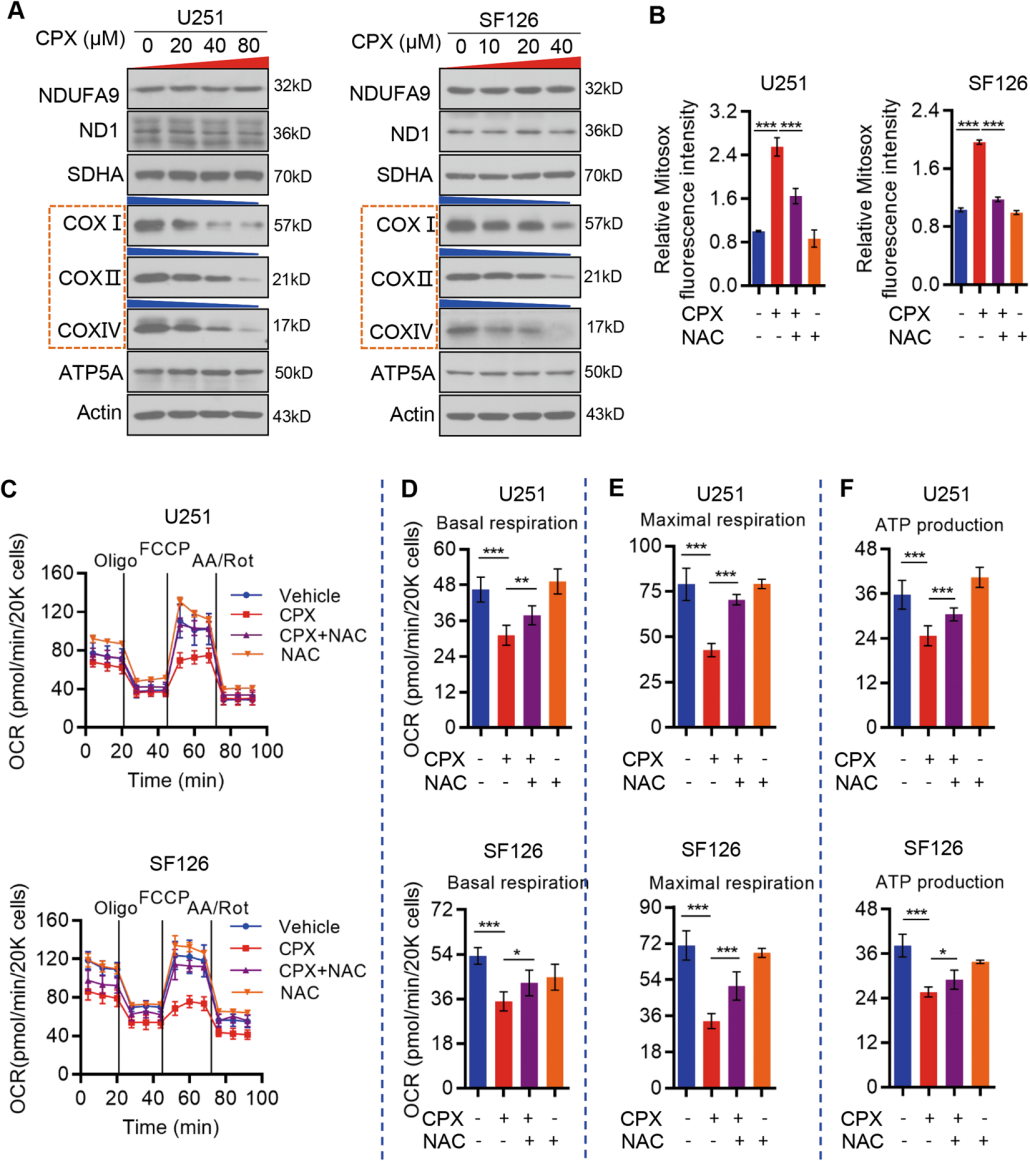
CPX impairs mitochondrial function. **A** U251 and SF126 cells were treated with CPX (0, 20, 40, and 80 µM for U251 cells; 0, 10, 20, and 40 µM for SF126 cells) for 48 h and indicated subunits of mitochondrial enzymatic complexes were detected by immunoblotting. **B** Mitochondrial ROS levels in U251 and SF126 cells following CPX, NAC, or combined treatments, were measured using a MitoSOX Red mitochondrial superoxide indicator kit. Data represent mean ± SD (*n* = 3; ***p < 0.001). **C** Mitochondrial respiration profiles of U251 (upper) and SF126 (lower) cells treated with (20 μM) or without CPX combined with NAC (10 mM). The representative graph represents the mean oxygen consumption rate (OCR) ± SD of six replicates. **D**–**F** Basal respiration (**D**), maximal respiration (**E**) and ATP production (**F**) of U251 and SF126 cells treated with or without CPX (40 µM) combined with NAC (10 mM). Data represent mean ± SD (*n* = 3; *p < 0.05; **p < 0.01; ***p < 0.001).

The majority of cellular energy, namely adenosine triphosphate (ATP), is produced in mitochondria via OXPHOS. Therefore, it is an essential cancer therapeutic strategy to target OXPHOS. To evaluate the effect of CPX on the mitochondrial respiratory chain, we first measured changes in the cellular oxygen consumption rate (OCR) in CPX-treated GBM cells. We observed a significant decrease of overall OCR in a dose-dependent manner in CPX-treated GBM cells (Supplementary Fig. S2a). All three indexes of mitochondrial respiration, basal and maximal respiration and ATP production, were significantly reduced after CPX treatment of GBM cells (Supplementary Fig. S2b–d), suggesting that CPX treatment caused an impairment of mitochondrial OXPHOS. Next, we determined the expression of a variety of subunits of enzymatic complexes of the mitochondrial OXPHOS system in GBM cells (U251 and SF126 cells). Immunoblotting demonstrated that the levels of cytochrome c oxidase (COX) subunits I (COX I), II (COX II), and IV (COX IV) were decreased upon CPX treatment in a dose-dependent manner. However, there was no significant difference in the protein expression of NADH:ubiquinone oxidoreductase subunit A9 (NDUFA9), NADH-ubiquinone oxidoreductase chain 1, succinate dehydrogenase (subunit A), or ATP synthase subunit alpha (Fig. 3A).

Since the majority of intracellular ROS are generated in the mitochondrial respiratory chain, the selective reduction of COX subunits led us to speculate that CPX-induced impairment of mitochondrial function may enhance ROS production. Indeed, mitochondrial ROS (mitoROS) levels were significantly increased upon CPX treatment, and treatment of GBM cells with NAC (a ROS scavenger) dramatically decreased ROS production (Fig. 3B). Moreover, NAC treatment rescued overall OCR reduction by CPX in GBM cells (Fig. 3C), as well as the three indexes of mitochondrial respiration, namely, basal and maximal respiration rate, and ATP production rate (Fig. 3D–F). Collectively, these data indicate that CPX impairs mitochondrial OXPHOS, thus leading to increased production of ROS in GBM cells.

### CPX and BTZ synergistically activate JNK/p38 MAPK signaling pathways

**Fig. 4:**
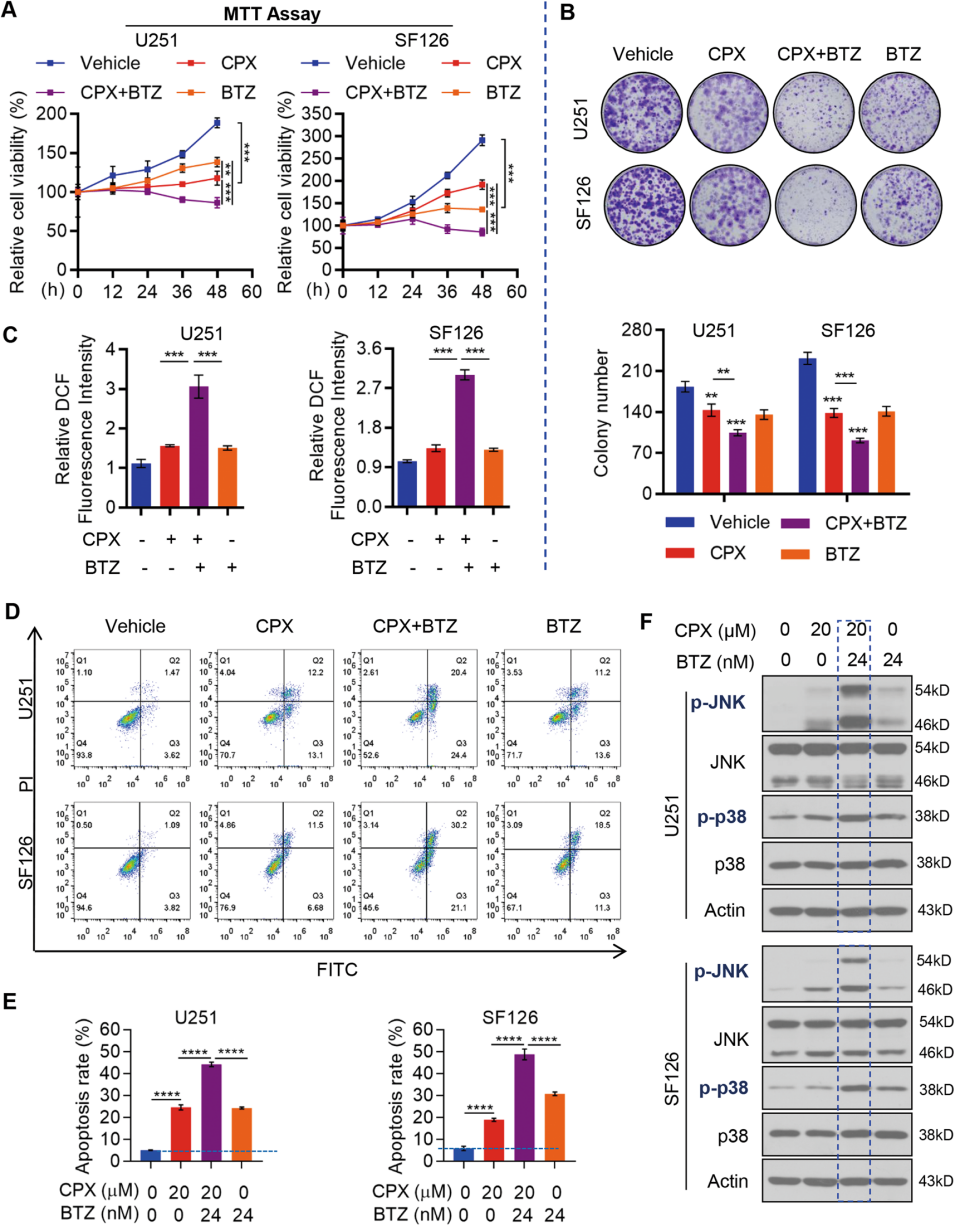
The combination of CPX and BTZ promotes apoptosis of GBM cells via activating the ROS-mediated JNK/p38 MAPK signaling. **A** MTT assays of U251 and SF126 cells in response to CPX (20 μM), BTZ (24 nM), or a combination of both drugs for the time indicated. Data represent mean ± SD (*n* = 3, **p < 0.01; ***p < 0.001). **B** Colony formation assays of U251 and SF126 cells treated with CPX (20 μM), BTZ (24 nM), or a combination of both drugs. Representative images (upper) and colony numbers (lower) from colony formation assays. Data represent mean ± SD of three independent experiments performed in triplicate (*n* = 3, **p < 0.01, ***p < 0.001). **C** U251 and SF126 cells were treated with CPX (20 μM), BTZ (24 nM), or a combination of both drugs, which promoted intracellular ROS production in U251 and SF126 cells measured by a ROS Assay Kit (DCFH-DA). Data are plotted as relative fluorescence intensities and are presented as mean ± SD (*n* = 3, ***p < 0.001). **D**, **E** U251 and SF126 cells were treated with CPX (20 μM), BTZ (24 nM), or a combination of both drugs for 24 h, and apoptosis was determined by flow cytometry. Representative images of apoptosis (**D**) and results from three independent experiments (**E**) (***p < 0.001). **F** Immunoblotting analysis of p38, p-p38, JNK, and p-JNK in U251 and SF126 cells treated with CPX (20 μM), BTZ (24 nM), or a combination of both drugs for 24 h.

BTZ has a cytotoxic effect in a variety of tumor cells^21^ and can reduce the therapeutic concentration of tumor necrosis factor-related apoptosis-inducing ligand, for treating GBM^22^. To determine whether BTZ reduces the concentration of CPX required for its effects in GBM cells, we combined BTZ with CPX to treat U251 and SF126 cells. Simultaneous treatment with CPX (20 μM) and BTZ (24 nM) for 24 h repressed the proliferation of GBM cells compared to the effect of CPX or BTZ alone (Fig. 4A and Supplementary Fig. S3) and also synergistically abolished colony formation (Fig. 4B). Correspondingly, both cellular ROS (Fig. 4C) and apoptosis (Fig. 4D, E) were significantly elevated when CPX and BTZ were used in combination. Correspondingly, phosphorylation of both JNK and p38 MAPK, which may be activated by ROS to trigger apoptosis^23^, was significantly increased by the combination of CPX and BTZ (Fig. 4F). These results suggest that the combination of CPX and BTZ promotes apoptosis of GBM cells by ROS-mediated activation of the JNK/p38 MAPK pathway.

### CPX and BTZ synergistically activate NF-κB signaling pathways

**Fig. 5:**
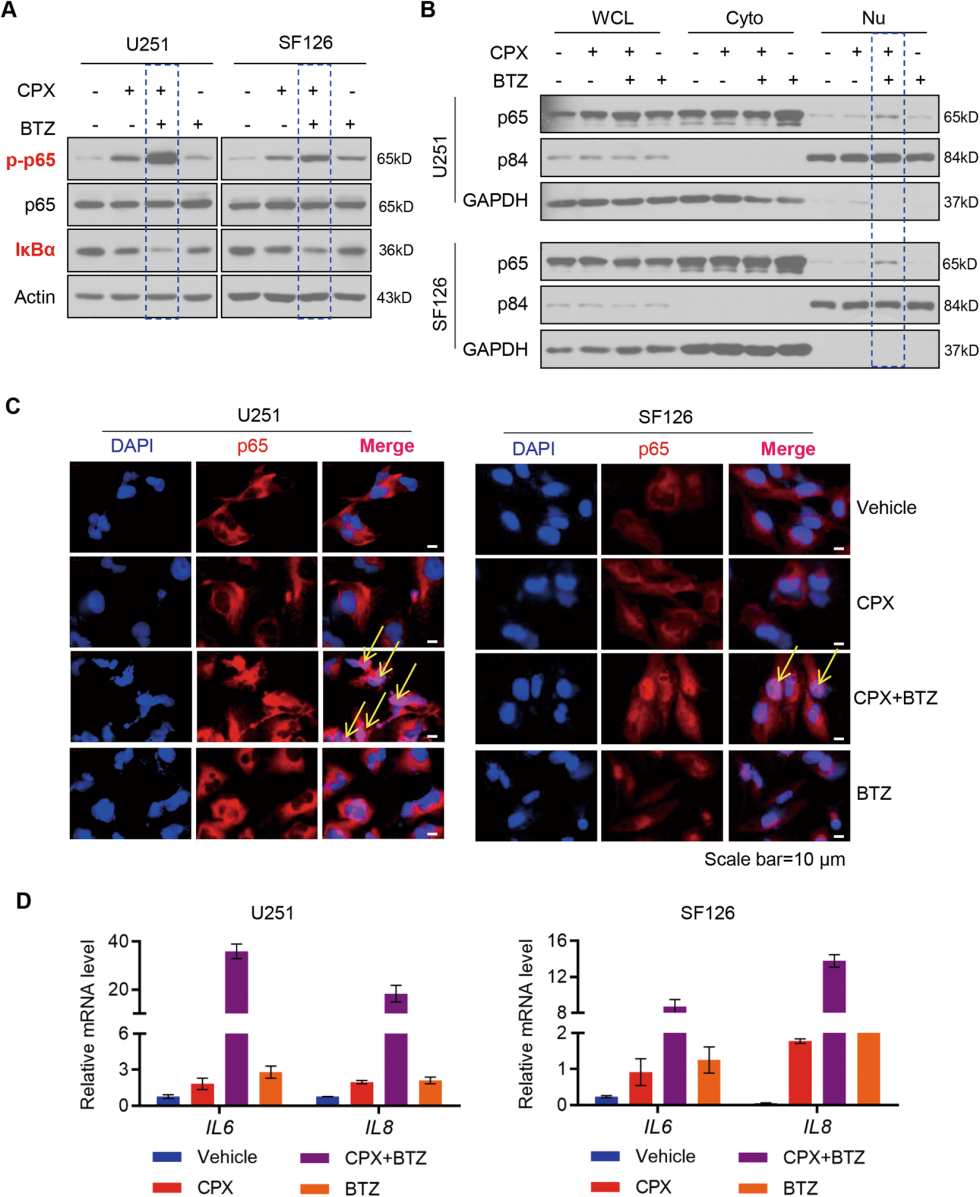
The combination of CPX and BTZ promotes the activation of NF-κB signaling to induce SASP. **A** Immunoblotting analysis of p-p65, p65, and IκBα in U251 and SF126 cells treated with vehicle (DMSO), CPX (20 μM), BTZ (24 nM), or a combination of both drugs for 24 h. **B** U251 and SF126 cells were treated with vehicle (DMSO), CPX (20 μM) and BTZ (24 nM) either alone or in combination for 24 h, and cytosolic (Cyto) or nuclear (Nu) fractions were isolated, followed by immunoblotting analysis with primary antibodies against p65, p84, and GAPDH. **C** Immunofluorescent analysis for p65 cellular localization of U251 and SF126 cells treated with vehicle (DMSO), CPX (20 μM), and BTZ (24 nM), either alone or in combination. **D** mRNA levels of the SASP markers, *IL6* and *IL8*, in U251 and SF126 cells treated with vehicle (DMSO), CPX (20 μM), and BTZ (24 nM), either alone or in combination.

To further investigate the synergistic effect as well as the molecular mechanism underlying this effect of CPX and BTZ in GBM cells, we examined the expression of NF-κB pathway-related proteins using immunoblotting assays. In the resting state, p65 and IκBs are in the form of inactive dimers in the cytosol. Upon induction by a variety of stimuli, IκBs are degradated and the released NF-κB dimers translocate to the nucleus, upon which NF-κB signaling is activated^24^. As shown in Fig. 5A, CPX or BTZ treatment increased p-p65 levels in GBM cells, whereas p65 expression was unchanged, and the NF-κB-signaling inhibitory protein, IκBα, decreased. Notably, combined treatment with CPX and BTZ led to a greatly enhanced up-regulation of p-p65 levels and down-regulation of IκBα protein expression in GBM cells (Fig. 5A). Furthermore, combined CPX and BTZ treatment synergistically promoted p65 nuclear translocation as evidenced by immunoblotting (Fig. 5B) and immunofluorescence (Fig. 5C and Supplementary Figs. S4a–d, S5a–d) analyses. Previous studies demonstrated that p65 enters the nucleus to induce the senescence-associated secretory phenotype (SASP) through regulating the expression of inflammation-related factors including interleukin 6 (IL6) and IL8^25^. Both *IL6* and *IL8* mRNAs were increased by CPX and BTZ alone, and synergistically by treatment of GBM cells with combined CPX and BTZ (Fig. 5D).

Therefore, we propose that combined CPX and BTZ treatment in GBM cells activates the NF-κB signaling pathway and induces the expression of *IL6* and *IL8* mRNAs.

### Combination of CPX and BTZ inhibits GBM tumor growth in vivo

**Fig. 6:**
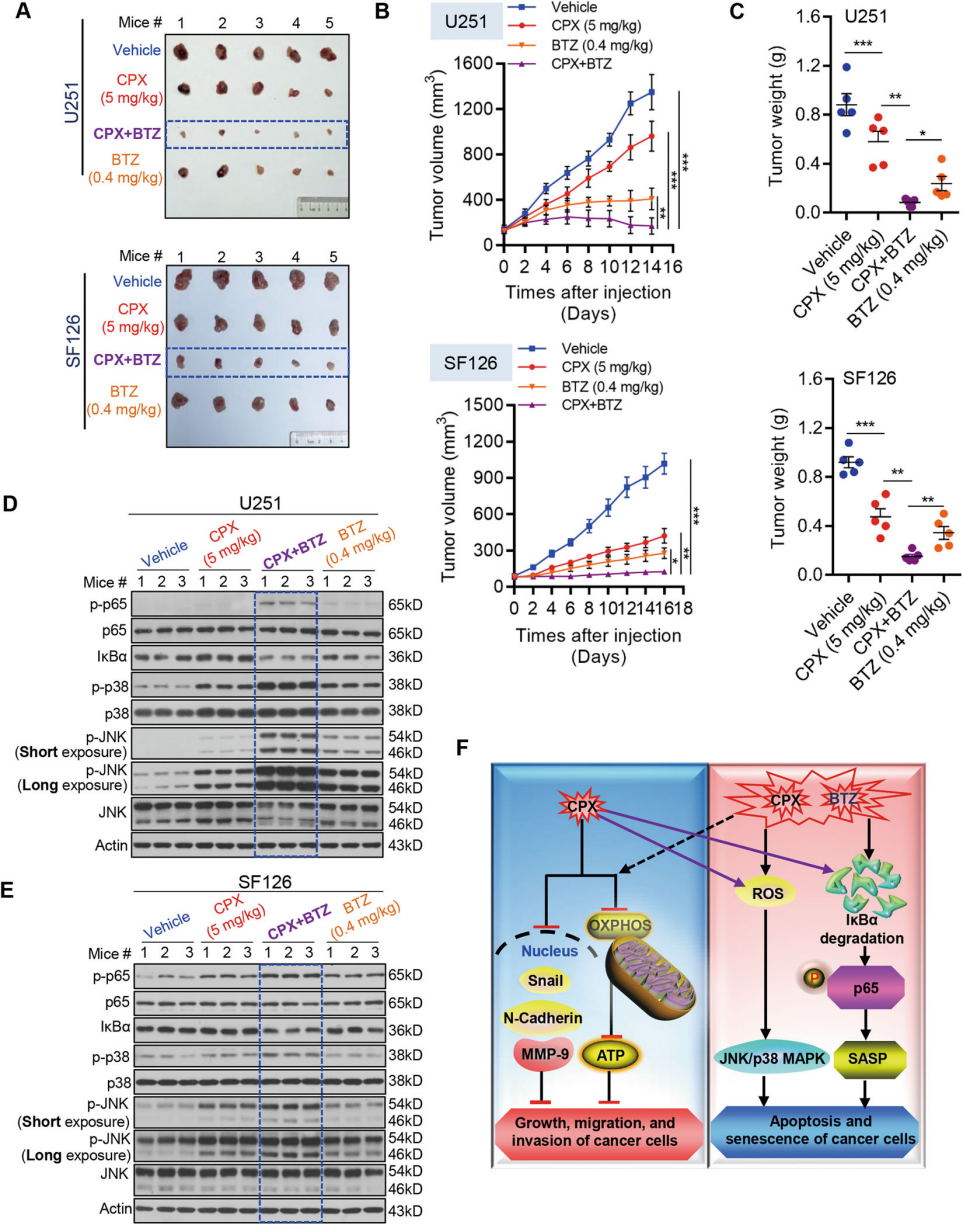
The combination of CPX and BTZ promotes inhibition of GBM cell growth in vivo. **A**–**C** Tumor-bearing nude mice of U251 cells (*n* = 5) were injected intraperitoneally twice daily for 14 consecutive days with physiological saline (0.9% NaCl), CPX (5 mg/kg), BTZ (0.4 mg/kg), or a combination of CPX and BTZ (5 mg/kg and 0.4 mg/kg, respectively). Mice were sacrificed on day 14, and their tumors were harvested and weighed. The dissected tumors are shown (**A**). Tumor volumes (**B**) and tumor weights (*n* = 5) of dissected tumors (**C**) from mice treated with 0.9% NaCl, CPX, BTZ, or a combination of CPX and BTZ. Data represent mean ± SD (*p < 0.05; **p < 0.01; ***p < 0.001). **D**, **E** Expression levels of p65, p-p65, IκBα, p38, p-p38, JNK, and p-JNK in tumor tissue lysates were examined by immunoblotting analysis. **F** Proposed mechanistic model of synergistic effects of CPX and BTZ inhibiting GBM cell growth, migration, and invasion.

We used the mouse xenograft model to investigate the inhibitory efficiency of combined CPX and BTZ on the growth of GBM cell tumors in vivo. U251 and SF126 cells were injected subcutaneously into the left flank of BALB/c nude mice (5 weeks old, *n* = 20). When the tumor volume reached ~100–200 mm^3^, tumor-bearing nude mice were randomly divided into four groups (receiving saline, CPX, BTZ, or combined CPX/BTZ), with five mice per group. Each mouse received an intraperitoneal injection of saline, or of drug(s) in saline solution, once a day for 14 consecutive days. Measurements of tumor volume (Fig. 6A, B) and weight (Fig. 6C) showed that the drugs reduced GBM cell growth in vivo, and that combined treatment with CPX and BTZ significantly repressed GBM cell growth in vivo compared to treatment with CPX or BTZ alone. None of the drugs caused obvious toxicity as assessed by body weight of the treated mice (Supplementary Fig. S6a, b). We also evaluated p-p65, IκBα, p-p38 and p-JNK levels in tumor sections via immunoblotting. Consistently, the combination of CPX and BTZ dramatically activated the JNK/p38 MAPK and NF-κB signaling pathways (Fig. 6D, E, and Supplementary Fig. S7a–d). Collectively, these results indicate that the combined treatment with CPX and BTZ significantly inhibited GBM cell growth in vivo, when compared with control saline or treatment with CPX or BTZ individually.

## Discussion

Epithelial–mesenchymal transition (EMT) is essential for cancer cells acquiring aggressive features and thus promoting metastasis and invasion of cancer cells. MMPs, which degrade and modify the extracellular matrix, also play critical roles in EMT to promote cancer metastasis.^26,27^ Accordingly, the present study shows that CPX significantly inhibited GBM cell growth, migration, and invasion via regulation of N-Cadherin-Snail-MMP9 signaling pathways in human GBM cell lines. Moreover, we showed that CPX caused mitochondrial dysfunction in GBM cells, and thus leding to mtROS accumulation and GBM cell death. Mitochondria, as the main source of cellular ATP, play an essential role in cellular metabolism, growth, survival, and apoptosis. Mitochondrial dysfunction and ROS are common features of many diseases, including cancer. There is increasing evidence that the majority of ROS production in mitochondria results from electron leakage of mitochondrial complex I and III^28,29^. These data, as well as the promising anti-GBM effect that we unveiled both in vitro and in vivo, strongly support the view that CPX may serve as a potential therapeutic strategy for GBM.

Preclinical studies have shown that the proteasome inhibitor, MG132, and BTZ can inhibit the proliferation of GBM^30,31^. In addition, BTZ was found to inhibit DNA repair enzyme O6-methylguanine methyltransferase (MGMT) to overcomes MGMT-mediated GBM chemoresistance. The proteasome inhibitor BTZ was also shown to enhance ROS production in mitochondria of various cancer cells and to cause cance cell apoptosis^32^. Recently, Wagner et al. reported a temporal variations in ROS generation in human glioblastoma T98G cells treated with BTZ^33^. Furthermore, while the EMT markers including Slug, MMP9, MMP2, CD44, N-Cadherin, and vimentin were suppressed by BTZ concomitant increased expression of E-Cadherin was detected in chondrosarcoma cells^34^. In the present study, we investigated whether it is feasible to use a combination of CPX and BTZ at lower doses without affecting their efficacies and, moreover, if such a combination may even synergistically enhance the resultant anti-tumor effects. Interestingly, we found that the proliferation and colony formation of U251 and SF126 cells were significantly inhibited via a CPX/BTZ combination, compared with results of either agent alone. It is well-known that increased intracellular ROS can activate the JNK/p38 MAPK pathway to induce apoptosis^23^. In our present study, we demonstrated that the combined treatment of CPX and BTZ promoted intracellular ROS accumulation and triggered activation of the JNK/p38 MAPK pathway, thereby inducing apoptosis of GBM cells.

NF-κB is a key regulator of diverse cellular processes such as differentiation, proliferation, and immune/inflammatory responses. The mammalian NF-κB family contains five members—p50/p105 (NF-κB1), p52/100 (NF-κB2), p65 (Rel-A), c-Rel, and Rel-B proteins—all of which associate with one another to form active homo- or hetero-dimers. NF-κB dimers form an inactive complex through interaction with IκB and are retained in the cytoplasm of senescent cells, since IκB masks the nuclear localization signal of NF-κB and blocks entry of the latter into the nucleus^35,36^. Disproportionate activation of NF-κB p65 signaling is closely associated with many chronic diseases, such as inflammatory and autoimmune diseases, and even cancers^24^. Therefore, NF-κB p65 signaling has been a key target for the discovery and development of novel drugs.

The role of NF-κB in tumorigenesis is complex, as in some mouse models the inhibition of NF-κB suppresses, whereas in others it facilitates, tumor development^37^. Previous studies have shown that the proapoptotic effect of BTZ in different cancers may, at least in part, be due to its inhibition of the NF-κB signaling pathway^38^. Bastian et al. have shown that the combination of BTZ and histone deacetylase inhibitors have synergistic activity in preclinical models of B-cell precursor acute lymphoblastic leukemia through activating NF-κB signaling^16^. The activation of NF-κB signaling contributes to the outcome of cancer therapy, especially via mediating chemotherapy-induced senescence^25,39,40^. In the present study, we provided evidence that the combination of CPX and BTZ led to the degradation of the NF-κB inhibitor, IκBα, and significantly enhanced activation of NF-κB signaling compared with these effects via CPX or BTZ alone, and dramatically upregulated NF-κB target genes (such as *IL-6* and *IL-8*) in GBM cells. Our data suggest that NF-κB supports senescence by activation of SASP. Further studies are required to elucidate whether CPX/BTZ-induced NF-κB activation represents a feedback of GBM cells to CPX/BTZ treatment.

Despite accumulating evidence for the synergistical effects of BTZ in combination of with several compounds to inhibit GBM cell growth, there is limited understanding on the underlying molecular mechanism. Notably, the combination of BTZ and SR9009, a synthetic pyrrole derivative that act as REV-ERB agonist, exhibits a significant synergistic effect on inhibiting GBM cell growth^41^. Interestingly, Vlachostergios et al found that BTZ may overcome MGMT-related chemoresistance of GBM cells to TMZ in a schedule-dependent manner through interfering NF-κB, MAPK, STAT3 and HIF-1α signaling pathway^19^. Furthermore, ursodeoxycholic acid, which is an endogenous bile acid existing in human bile, synergizes with BTZ to inhibit GBM progression through promoting ER stress related apoptosis^42^. Here we describe a promising therapeutic combination for treatment of GBM. Our results demonstrate that CPX plays an anti-tumor role in GBM cells. Moreover, the combined use of CPX and BTZ also synergistically activates the JNK/p38 MAPK pathway to induce apoptosis. CPX/BTZ co-treatment also activated the NF-κB pathway to promote p65 entry into the nucleus, thereby leading to inflammation-associated senescence of GBM cells. Taken together, we propose a mechanistic model of synergistic effects of CPX and BTZ against GBM cells via simultaneously enhancing JNK/p38 MAPK and NF-κB signaling (Fig. 6F). CPX/BTZ co-treatment also activated the NF-κB pathway to promote p65 entry into the nucleus, thereby leading to inflammation-associated senescence of GBM cells. These findings reveal a potentially rapid and cost-effective strategy to re-purpose CPX and BTZ for co-treatment of GBM. Novel drug-delivery strategies may be required to enable CPX and BTZ to bypass the blood-brain barrier, or it may prove possible to deliver CPX and BTZ directly into intracranial tumors. Alternatively, these compounds may serve as leads for development of drugs with superior properties.

## Supplementary information

Supplementary Figure Legends

Supplementary Figure 1

Supplementary Figure 2

Supplementary Figure 3

Supplementary Figure 4A

Supplementary Figure 4B

Supplementary Figure 4C

Supplementary Figure 4D

Supplementary Figure 5A

Supplementary Figure 5B

Supplementary Figure 5C

Supplementary Figure 5D

Supplementary Figure 6

Supplementary Figure 7

Supplementary Table 1

Supplementary Table 2
