# Supplementary material for: Ciclopirox and bortezomib synergistically inhibits glioblastoma multiforme growth via simultaneously enhancing JNK/p38 MAPK and NF-κB signaling
Source: Cell Death Dis. 2021 Mar 5;12(3):251. doi: 10.1038/s41419-021-03535-9 (PMC7935936; doi:10.1038/s41419-021-03535-9)
Supplement: Supplementary file 1 — Supplementary Figure Legends [file 41419_2021_3535_MOESM1_ESM.docx]

**Supplementary Figure 1**. **CPX suppresses GBM cellular growth in vitro. a** Proliferation of U251 and SF126 cells treated with CPX for four days was measured by CCK-8 cellular proliferation and cytotoxicity assay kits. Data represent mean ± SD (n = 3, ^***^p < 0.001). **b** The IC_50_ values of CPX for the GBM cell lines, A172 and U118, were assessed by MTT assays. **c,d** A172 (left panel) and U118 (right panel) cells were treated with CPX for four days, and cellular proliferation was determined by MTT (**c**) and CCK-8 (**d**) assays, respectively. Data represent means ± SDs (n = 3; ^***^p < 0.001). **e,f** Colony formation assays of A172 and U118 cells treated with increasing concentrations of CPX for 48 h. Representative images of colony formation assays are shown (**e**). Colony number counts (**f**) are presented as the mean ± SD (n = 3, ^***^p < 0.001).

**Supplementary Figure 2.** **CPX impairs mitochondrial OXPHOS. a** Overall mitochondrial OCR curve of U251, SF126, A172, and U118 cells treated with CPX for 48 h. **b–d** Basal (**b**), maximal respiration (**c**) and ATP production were evaluated (**d**). Data represent mean ± SD (n=6; ^***^p < 0.001).

**Supplementary Figure 3. Relative cell viability assay.** CCK-8 assays of U251 and SF126 cells in response to CPX (20 μM), BTZ (24 nM), or a combination of both drugs for the time indicated. Data represent mean ± SD (n=3, ^**^p < 0.01, ^***^p < 0.001).

**Supplementary Figure 4.** **3D volume view and 3D section view of single cells labelled with DPAI and p65 in U251 cells**. U251 cells were treated with vehicle (DMSO) **(a)**, CPX (20 μM) **(b)**, the combination of CPX (20 μM) and BTZ (24 nM) **(c)**, and BTZ (24 nM) **(d)** for 24 h. **3D confocal microscopy:** The cell was imaged under Leica SP8 confocal microscopy equipped with white laser. Nucleus was labeled with DAPI (excitation = 405 nm, emission = 430 – 450 nm); p65 was immunofluorescently labeled by Alexa Fluor Plus 555 secondary antibody (excitation = 555 nm, emission = 570 – 600 nm). The 3D micrographs were taken under Leica Sequential model and reconstructed by Huygens Professional with authorized license.

**Supplementary Figure 5. 3D volume view and 3D section view of single cells labelled with DPAI and p65 in SF126 cells**. SF126 cells were treated with vehicle (DMSO) **(a)**, CPX (20 μM) **(b)**, the combination of CPX (20 μM) and BTZ (24 nM) **(c)**, and BTZ (24 nM) **(d)** for 24 h. **3D confocal microscopy:** The cell was imaged under Leica SP8 confocal microscopy equipped with white laser. Nucleus was labeled with DAPI (excitation = 405 nm, emission = 430 – 450 nm); p65 was immunofluorescently labeled by Alexa Fluor Plus 555 secondary antibody (excitation = 555 nm, emission = 570 – 600 nm). The 3D micrographs were taken under Leica Sequential model and reconstructed by Huygens Professional with authorized license.

**Supplementary Figure 6. The potential toxicity of CPX combined with BTZ in mice.** Changes in body weight after 0.9% NaCl, CPX, BTZ, or CPX/BTZ co-treatment in either a U251 **(a)** or SF126 **(b)** mouse xenograft model. Data represent mean ± SD (n=5; ns, not significant).

**Supplementary Figure 7. The combination of CPX and BTZ dramatically activates the JNK/p38 MAPK and NF-κB signaling pathways *in vivo*. a-d** Quantification of IκBα **(a)**, p-p65 **(b)**, p-p38 **(c)** and p-JNK **(d)** protein levels in tumor tissues following 0.9% NaCl, CPX, BTZ, or a CPX/BTZ co-treatment in a U251 and SF126 mouse xenograft model. Data represent mean ± SD (n = 3; ^*^p < 0.05, ^**^p < 0.01, ^***^ p < 0.001).
