# Supplementary figures and images for: Ciclopirox and bortezomib synergistically inhibits glioblastoma multiforme growth via simultaneously enhancing JNK/p38 MAPK and NF-κB signaling
Source: Cell Death Dis. 2021 Mar 5;12(3):251. doi: 10.1038/s41419-021-03535-9 (PMC7935936; doi:10.1038/s41419-021-03535-9)

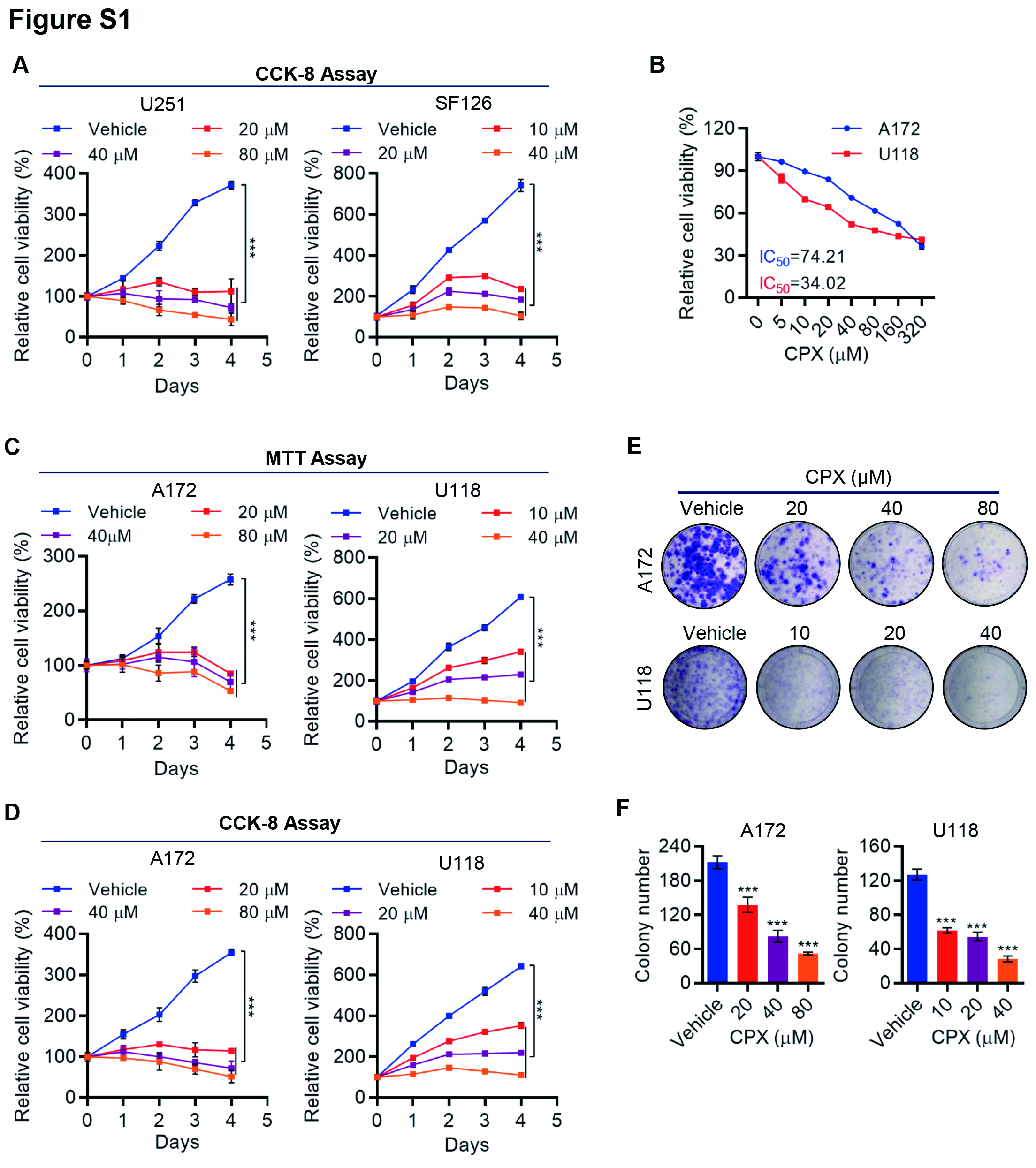

Supplement: Supplementary file 2 — Supplementary Figure 1 [file 41419_2021_3535_MOESM2_ESM.tif]

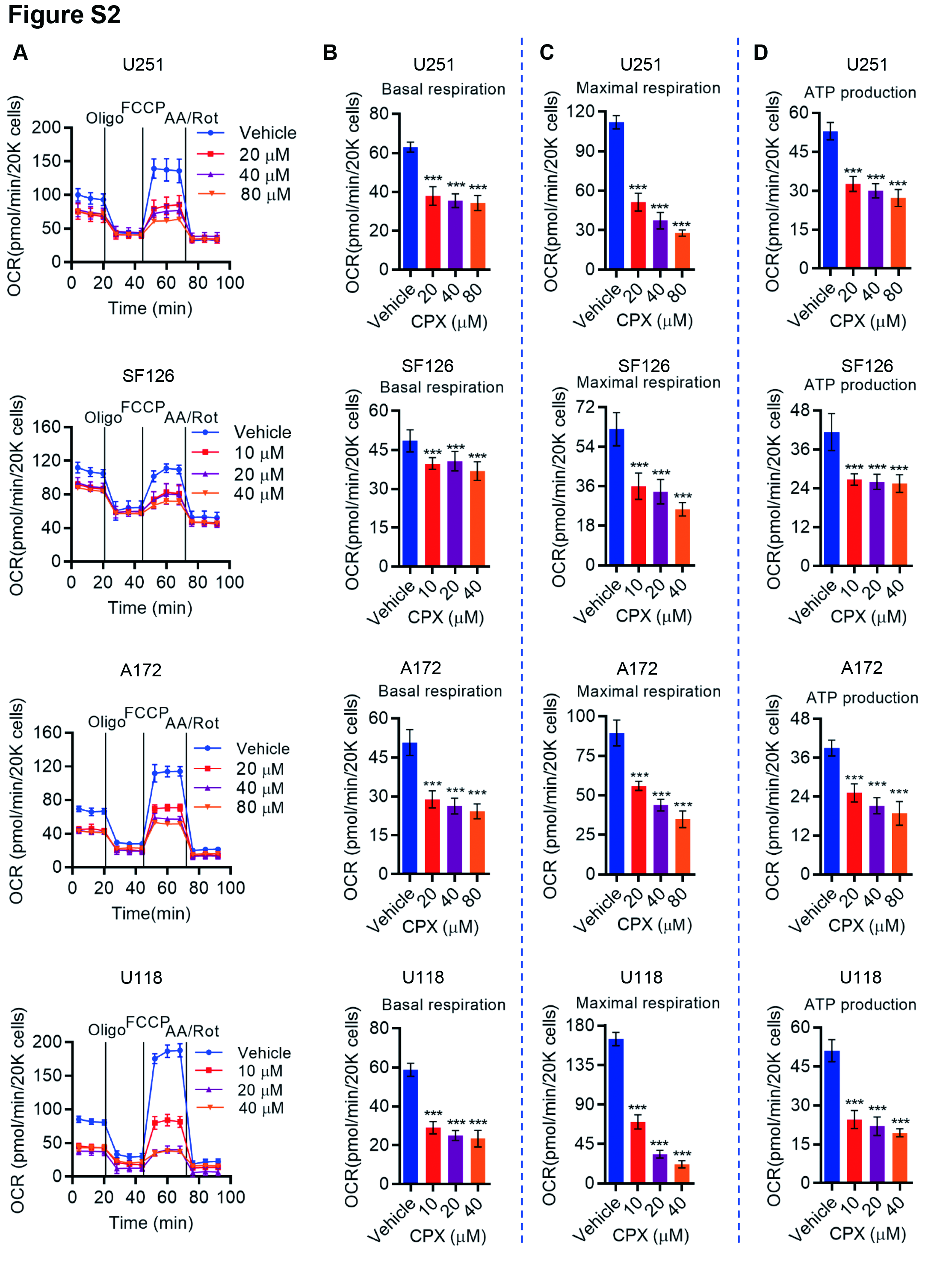

Supplement: Supplementary file 3 — Supplementary Figure 2 [file 41419_2021_3535_MOESM3_ESM.tif]

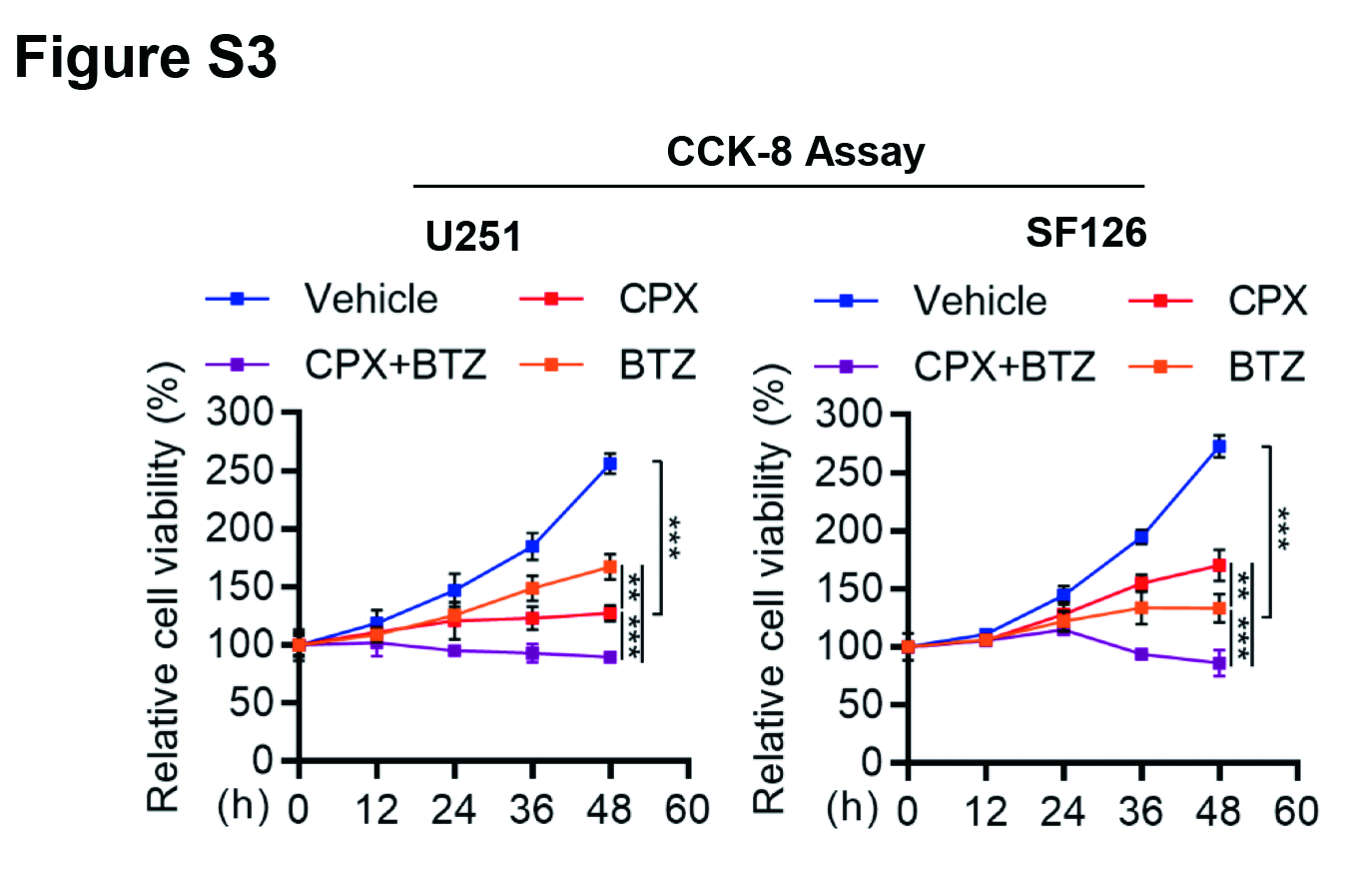

Supplement: Supplementary file 4 — Supplementary Figure 3 [file 41419_2021_3535_MOESM4_ESM.tif]

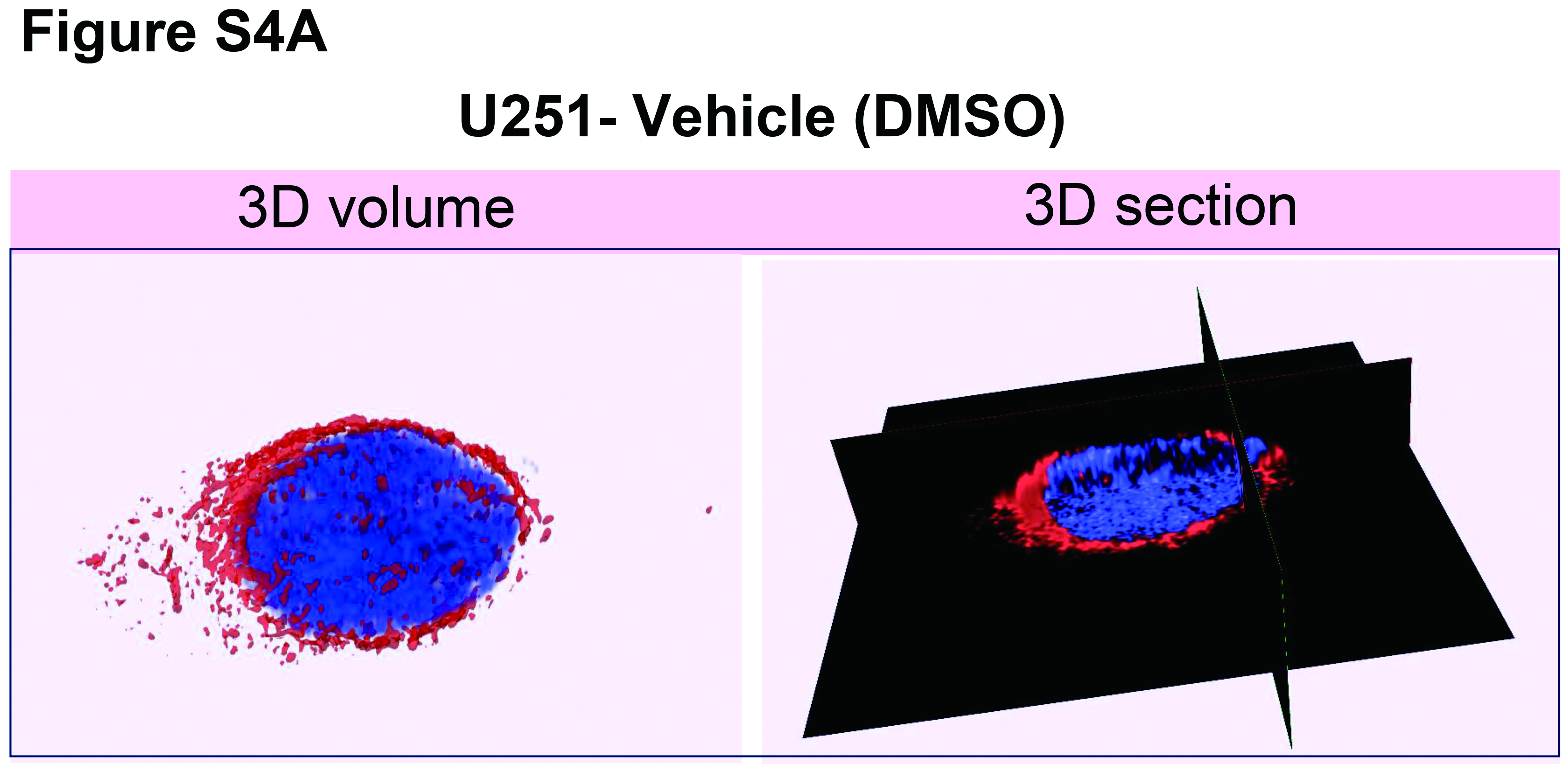

Supplement: Supplementary file 5 — Supplementary Figure 4A [file 41419_2021_3535_MOESM5_ESM.tif]

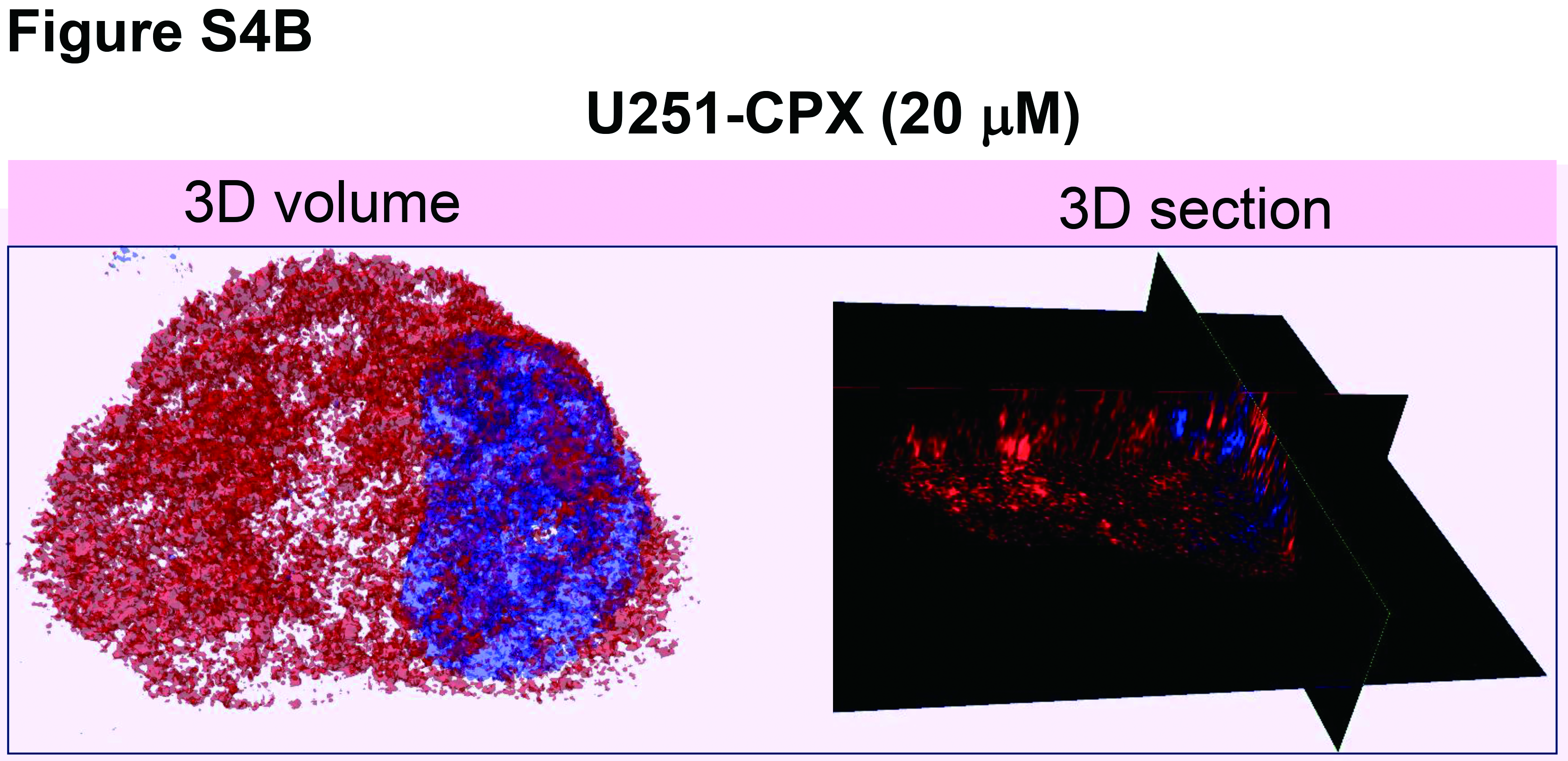

Supplement: Supplementary file 6 — Supplementary Figure 4B [file 41419_2021_3535_MOESM6_ESM.tif]

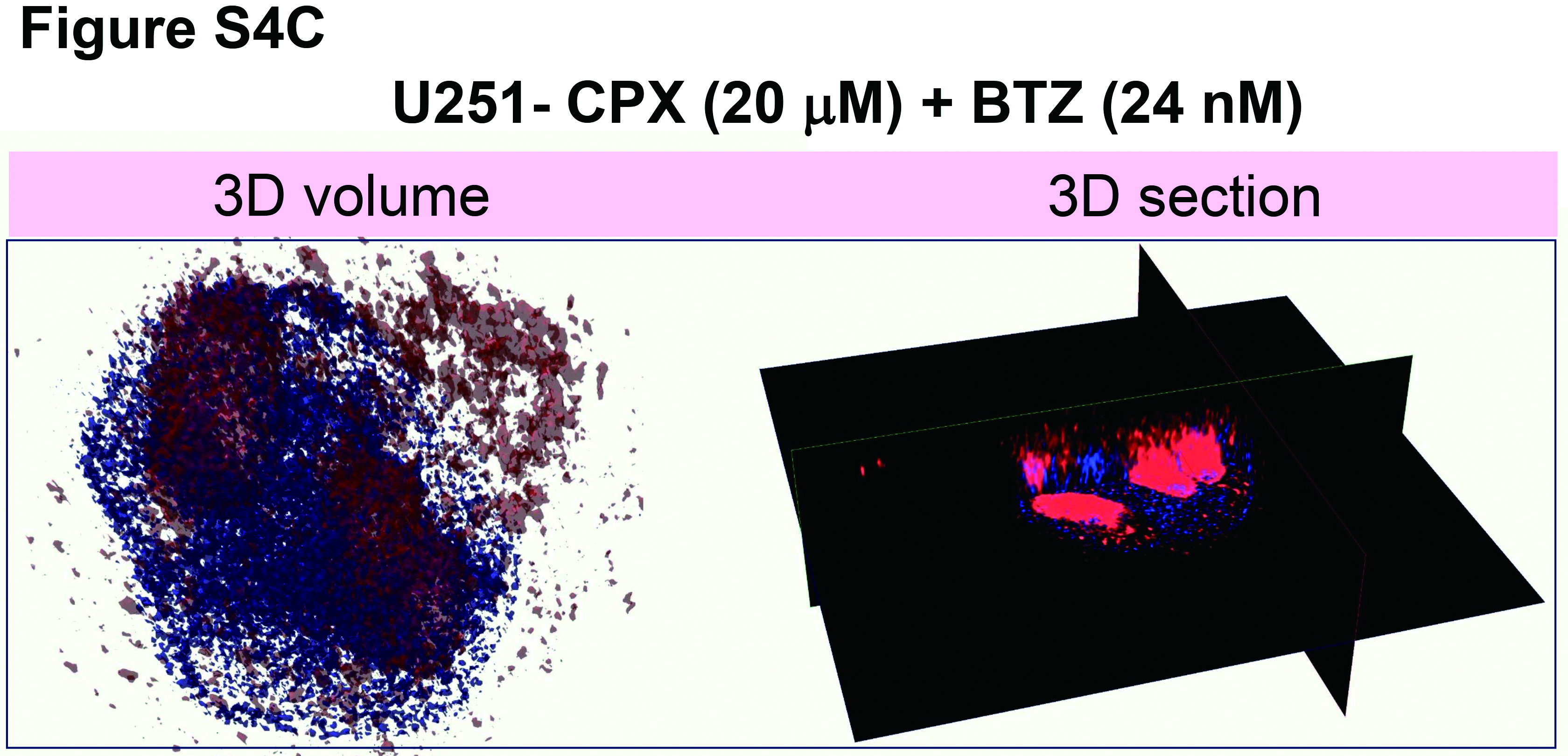

Supplement: Supplementary file 7 — Supplementary Figure 4C [file 41419_2021_3535_MOESM7_ESM.tif]

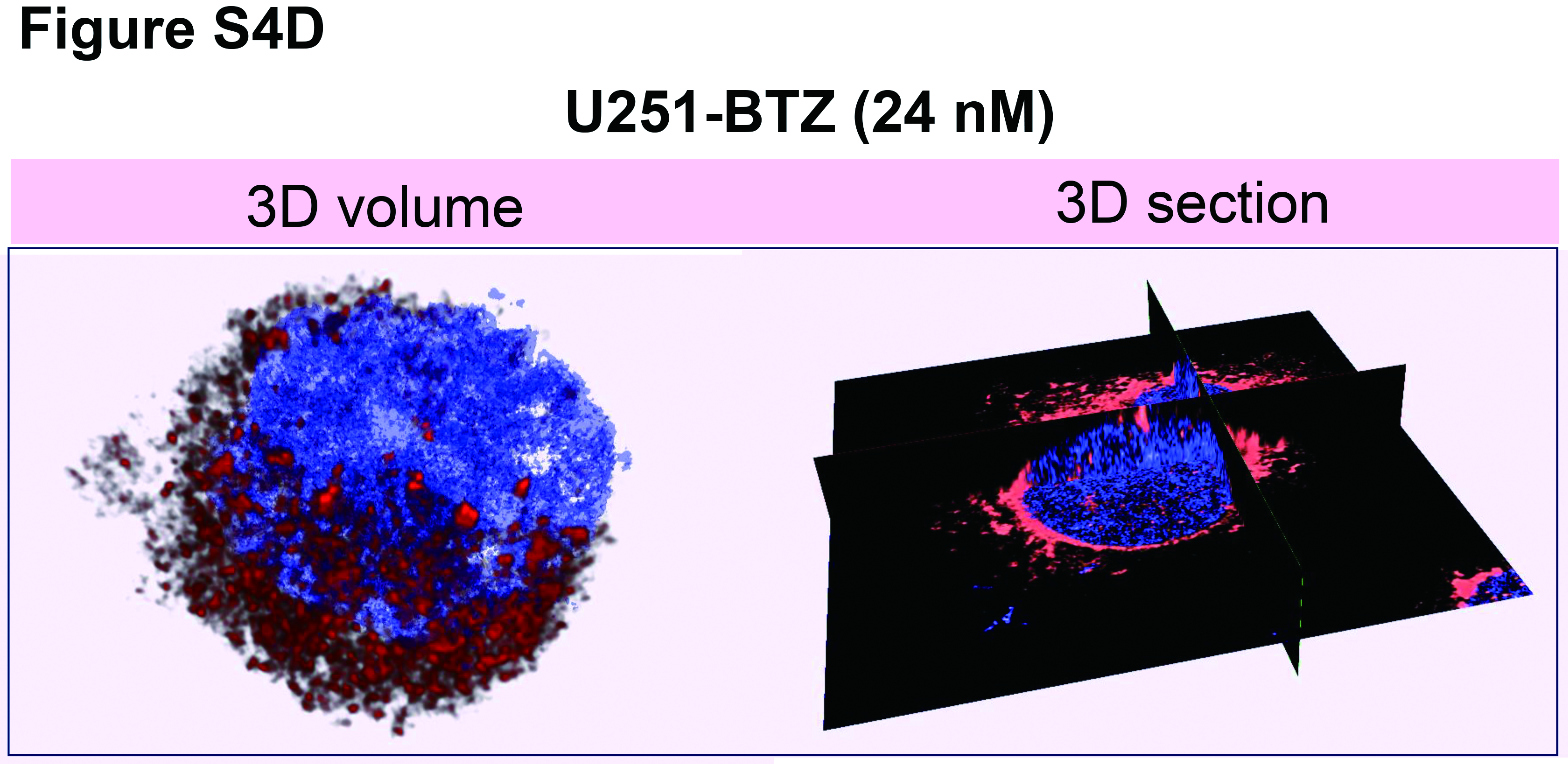

Supplement: Supplementary file 8 — Supplementary Figure 4D [file 41419_2021_3535_MOESM8_ESM.tif]

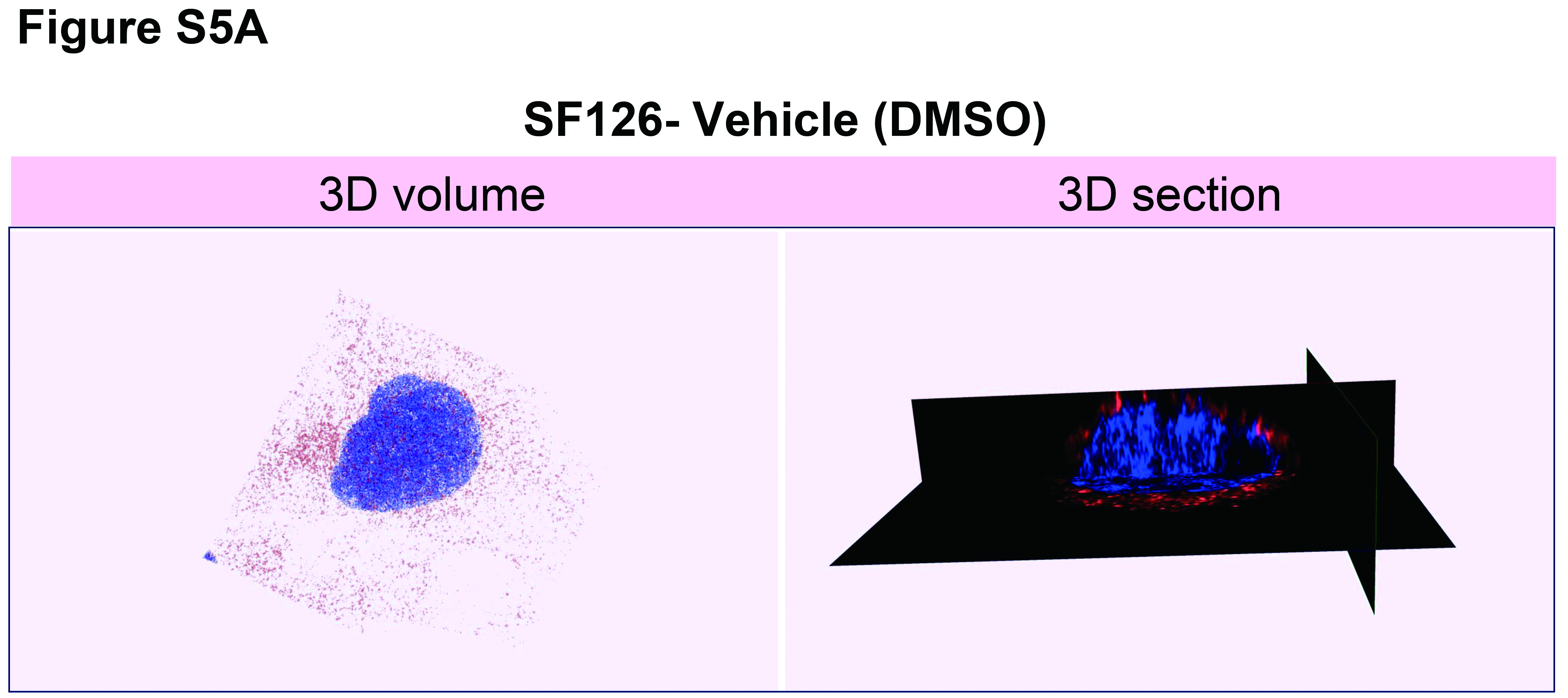

Supplement: Supplementary file 9 — Supplementary Figure 5A [file 41419_2021_3535_MOESM9_ESM.tif]

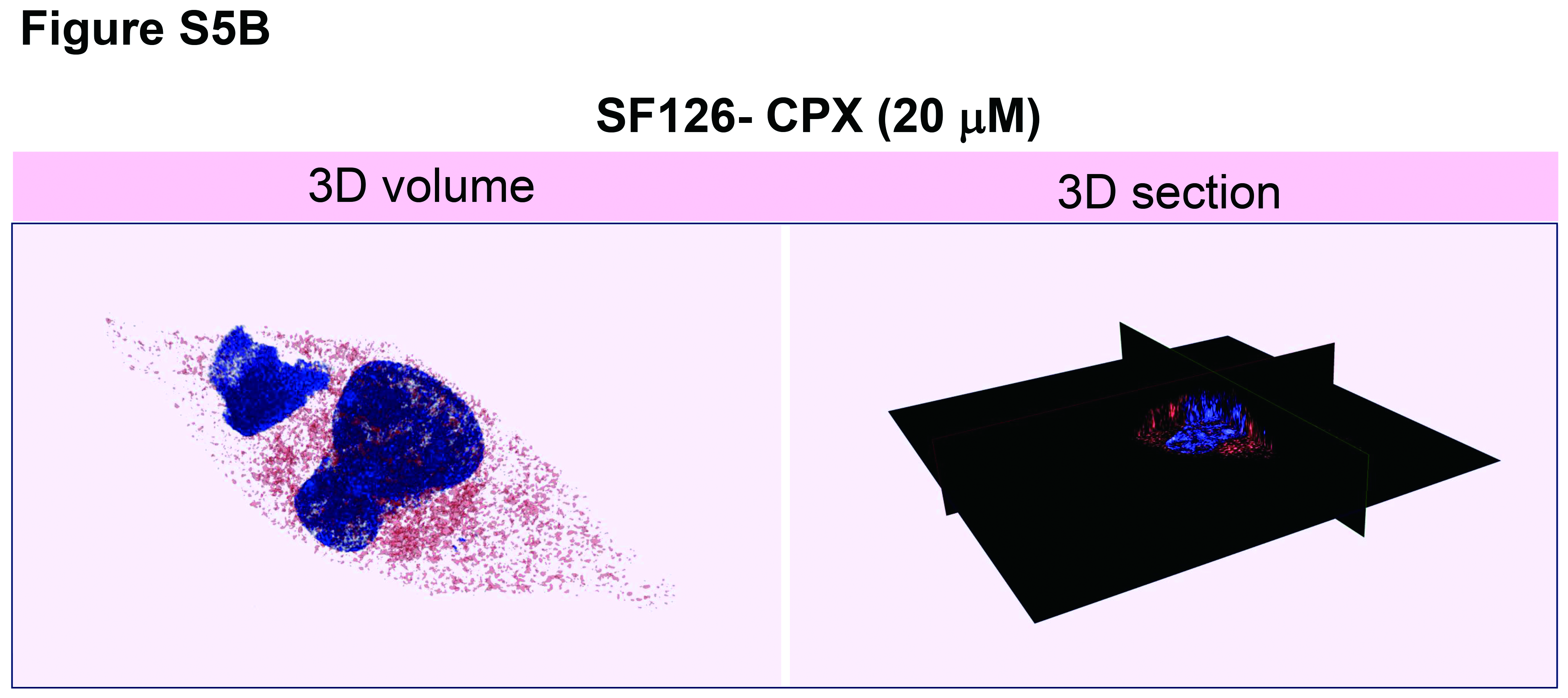

Supplement: Supplementary file 10 — Supplementary Figure 5B [file 41419_2021_3535_MOESM10_ESM.tif]

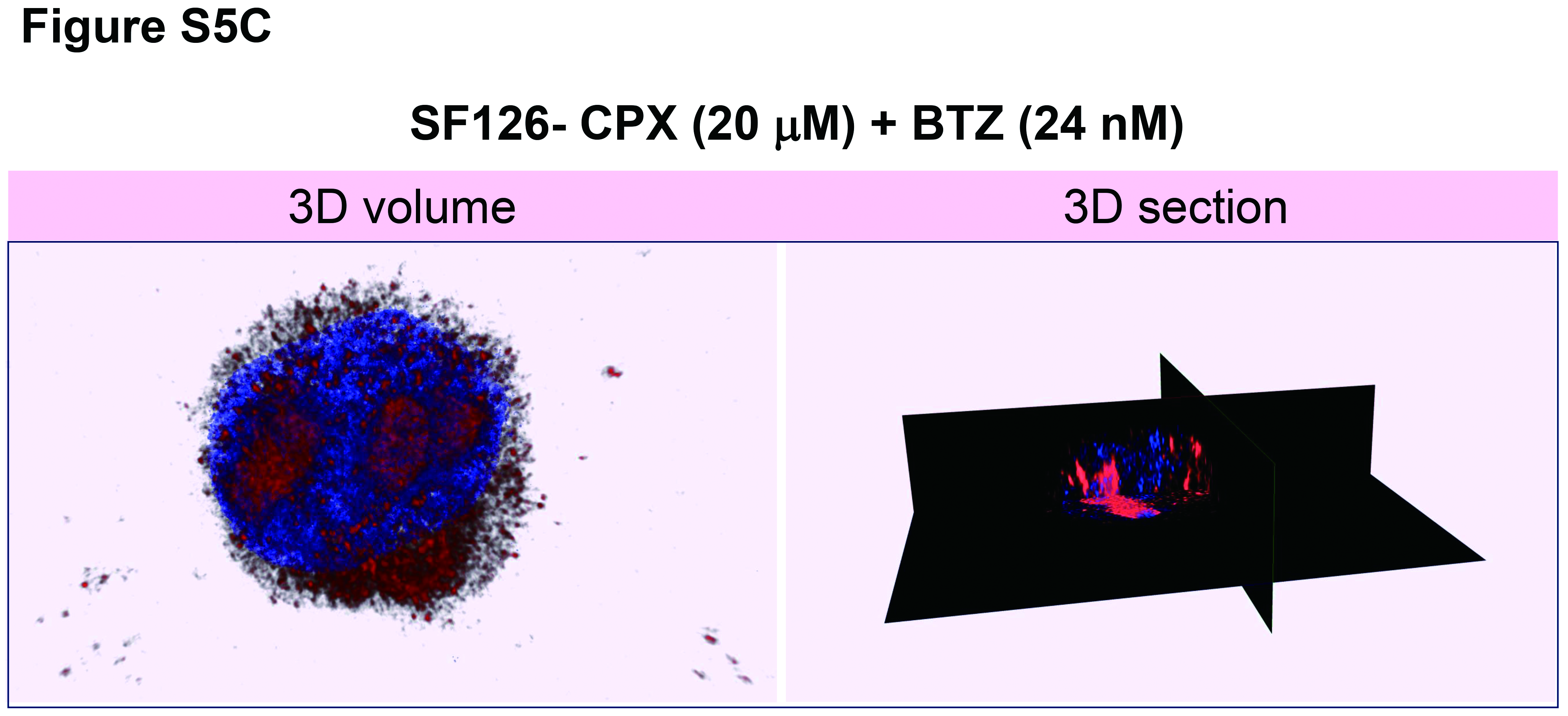

Supplement: Supplementary file 11 — Supplementary Figure 5C [file 41419_2021_3535_MOESM11_ESM.tif]

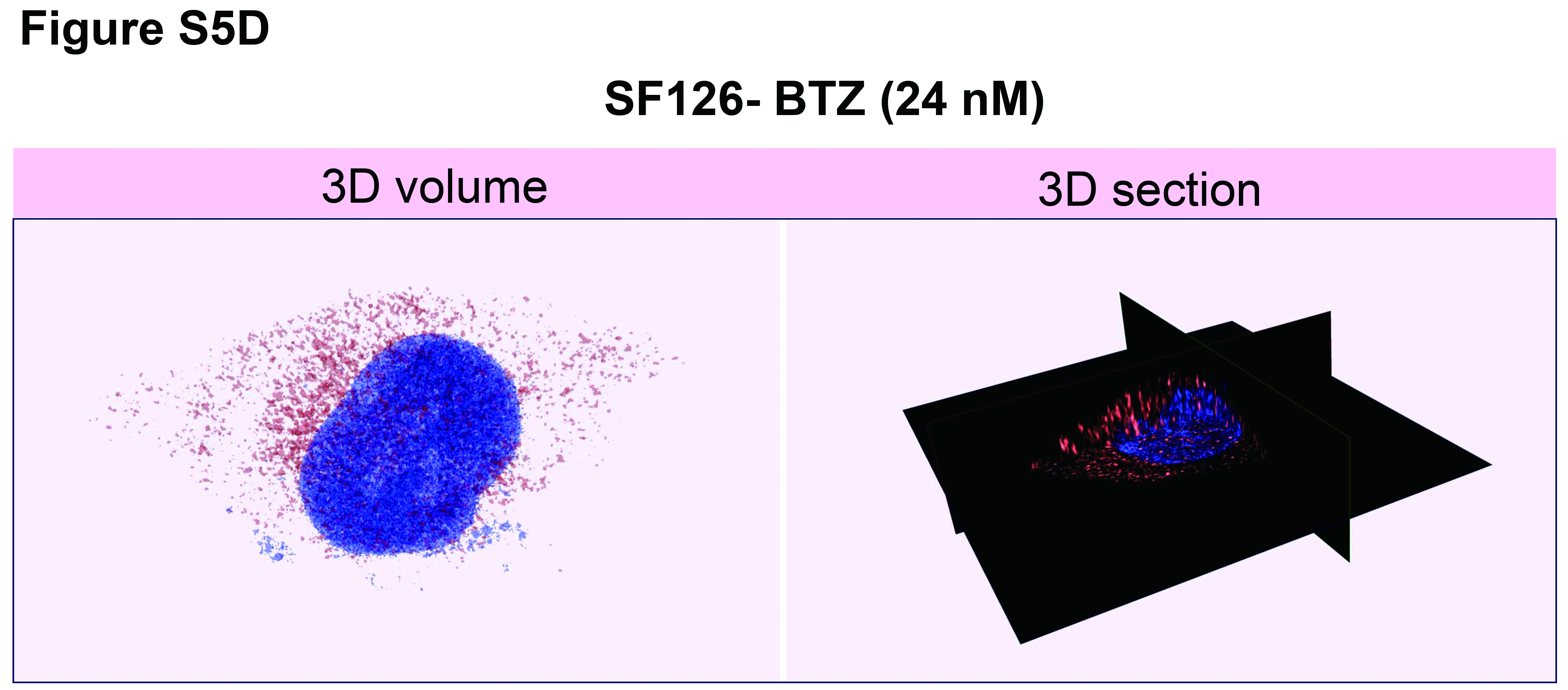

Supplement: Supplementary file 12 — Supplementary Figure 5D [file 41419_2021_3535_MOESM12_ESM.tif]

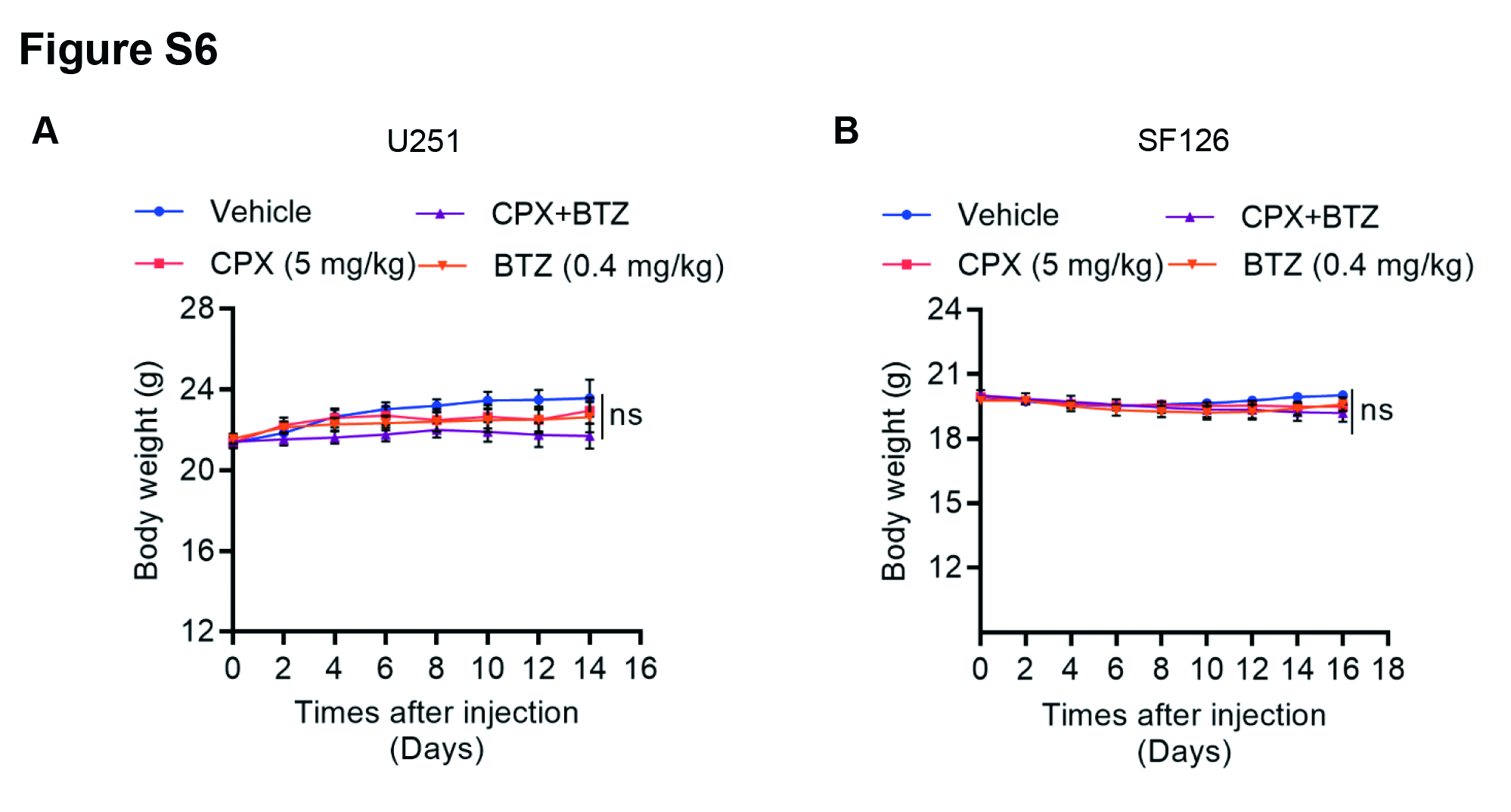

Supplement: Supplementary file 13 — Supplementary Figure 6 [file 41419_2021_3535_MOESM13_ESM.tif]

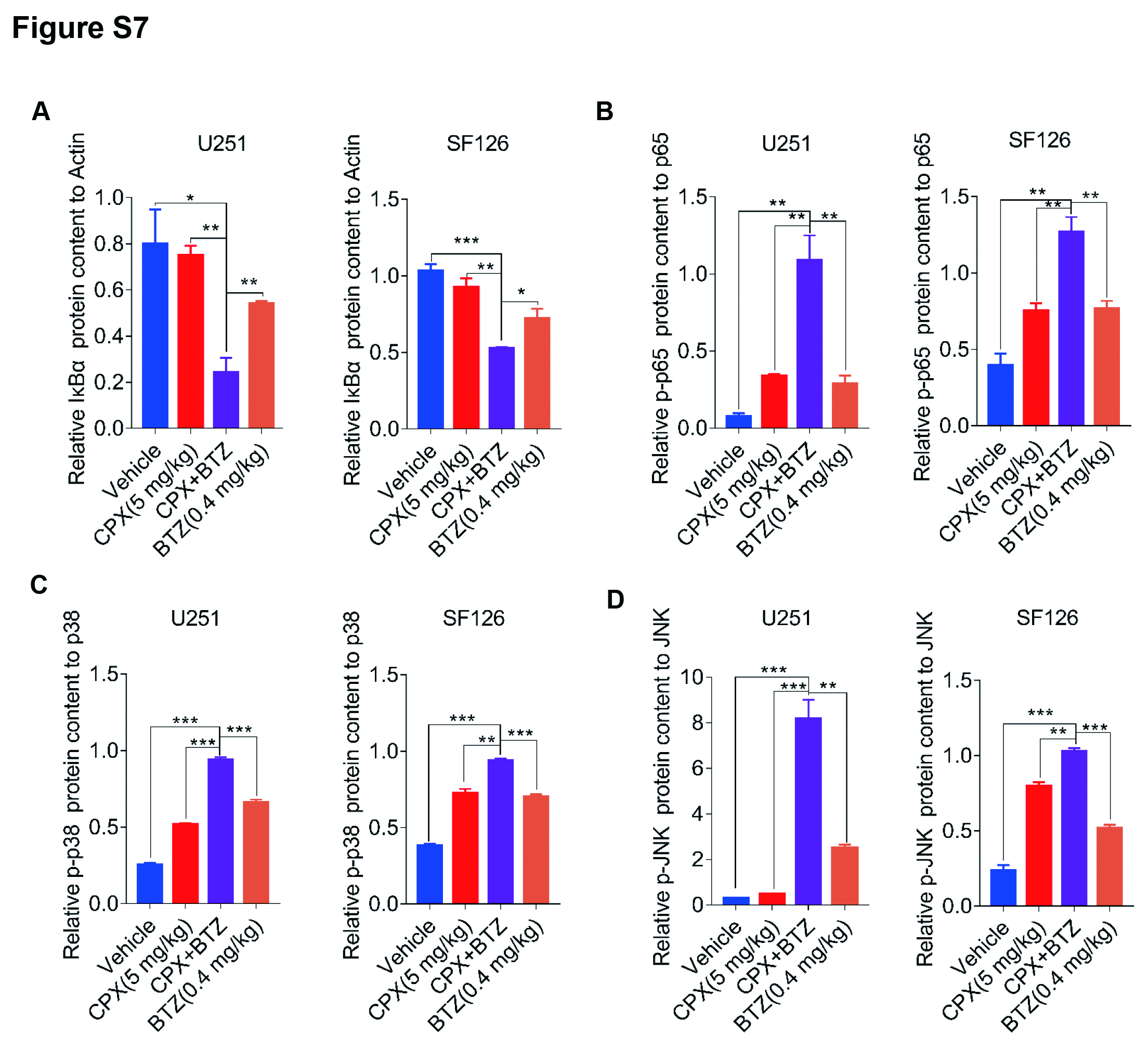

Supplement: Supplementary file 14 — Supplementary Figure 7 [file 41419_2021_3535_MOESM14_ESM.tif]
