# Supplementary material for: Ciclopirox and bortezomib synergistically inhibits glioblastoma multiforme growth via simultaneously enhancing JNK/p38 MAPK and NF-κB signaling
Source: Cell Death Dis. 2021 Mar 5;12(3):251. doi: 10.1038/s41419-021-03535-9 (PMC7935936; doi:10.1038/s41419-021-03535-9)
Supplement: Supplementary file 15 — Supplementary Table 1 [file 41419_2021_3535_MOESM15_ESM.docx]

**Supplementary Table 1. Primers used for qRT-PCR.**

| **Gene** | **Forward primer** | **Reverse primer** |
| --- | --- | --- |
| ***IL6***  ***IL8***  ***GAPDH*** | 5’ CTTCGGTCCAGTTGCCTTCTC 3’  5’ CTCTTGGCAGCCTTCCTGA3’  5’GACTCATGACCACAGTCCATGC3’ | 5’ATTCGTTCTGAAGAGGTGAGTGG 3’  5’ TTTCTGTGTTGGCGCAGTGT3’  5’CAGGTCAGGTCCACCACTGA3’ |
