# Supplementary material for: Ciclopirox and bortezomib synergistically inhibits glioblastoma multiforme growth via simultaneously enhancing JNK/p38 MAPK and NF-κB signaling
Source: Cell Death Dis. 2021 Mar 5;12(3):251. doi: 10.1038/s41419-021-03535-9 (PMC7935936; doi:10.1038/s41419-021-03535-9)
Supplement: Supplementary file 16 — Supplementary Table 2 [file 41419_2021_3535_MOESM16_ESM.docx]

**Supplementary Table 2. Details of the antibodies used for Western-blot analyses.**

| **Antibodies** | **SOURCE** | **Cat #** |
| --- | --- | --- |
| Anti-SDHA | Abcam | ab14715 |
| Anti-COXI  Anti-COXII  Anti-COXIV  Anti-ND1  Anti-ATP5A  Anti-Lamin B1  Anti-p84  Anti-p-p65  Anti-p-JNK  Anti-JNK  Anti-p-p38  Anti-p38  Anti-NDUFA9  Anti-p65  Anti-GAPDH  Anti-Actin  Anti-N-Cadherin  Anti-MMP9  Anti-IκBα  Anti-Snail | Abcam  Abcam  Abcam  Abcam  Abcam  Abcam  Abcam  Cell signaling Technology  Cell signaling Technology  Cell signaling Technology  Cell signaling Technology  Cell signaling Technology  Proteintech  Proteintech  Abmart  Abmart  BD Biosciences  Abclonal  Abclonal  Novus Biologicals | ab14705  ab110258  ab140643  ab181848  ab14748  ab16048  ab131268  3033  4668  9252  9211  9212  20312-1-AP  10745-1-AP  P30008  P30002  610920  A0289  A11397  NBP2-50300 |
